# MCM2-regulated functional networks in lung cancer by multi-dimensional proteomic approach

**DOI:** 10.1038/s41598-017-13440-x

**Published:** 2017-10-16

**Authors:** Chantal Hoi Yin Cheung, Chia-Lang Hsu, Kai-Pu Chen, Siao-Ting Chong, Chang-Hsun Wu, Hsuan-Cheng Huang, Hsueh-Fen Juan

**Affiliations:** 1 Institute of Molecular and Cellular Biology, National Taiwan University, Taipei, 10617 Taiwan; 2 Department of Life Science, National Taiwan University, Taipei, 10617 Taiwan; 3 Graduate Institute of Biomedical Electronics and Bioinformatics, National Taiwan University, Taipei, 10617 Taiwan; 4 Institute of Biomedical Informatics, Center for Systems and Synthetic Biology, National Yang-Ming University, Taipei, 11221 Taiwan

## Abstract

DNA replication control is vital for maintaining genome stability and the cell cycle, perhaps most notably during cell division. Malignancies often exhibit defective minichromosome maintenance protein 2 (MCM2), a cancer proliferation biomarker that serves as a licensing factor in the initiation of DNA replication. MCM2 is also known to be one of the ATPase active sites that facilitates conformational changes and drives DNA unwinding at the origin of DNA replication. However, the biological networks of MCM2 in lung cancer cells via protein phosphorylation remain unmapped. The RNA-seq datasets from The Cancer Genome Atlas (TCGA) revealed that MCM2 overexpression is correlated with poor survival rate in lung cancer patients. To uncover MCM2-regulated functional networks in lung cancer, we performed multi-dimensional proteomic approach by integrating analysis of the phosphoproteome and proteome, and identified a total of 2361 phosphorylation sites on 753 phosphoproteins, and 4672 proteins. We found that the deregulation of MCM2 is involved in lung cancer cell proliferation, the cell cycle, and migration. Furthermore, HMGA1^S99^ phosphorylation was found to be differentially expressed under MCM2 perturbation in opposite directions, and plays an important role in regulating lung cancer cell proliferation. This study therefore enhances our capacity to therapeutically target cancer-specific phosphoproteins.

## Introduction

Lung cancer is the leading cause of cancer-related mortality accounting for about 27% of all cancer deaths per year^1^. Despite the introduction of improved treatments, the overall 5-year survival rate of lung cancer patients remains low (<15%), and less than 7% of patients survive 10 years following diagnosis across all stages of lung cancer^2^. Elucidation of the biological network perturbations between cancer-related proteins is one promising route to alter this mortality trend^3^. The deregulation of protein interactions in DNA replication, proliferation, and the cell cycle are some of the key factors involved in cancer development and progression^4,5^. A new perspective on cancer progression has been suggested, wherein the genes driving cell proliferation induce DNA replication stress and promote further genomic instability^6^. Therefore, eukaryotic replication factors such as topoisomerases and DNA ligases have emerged as potential chemotherapeutic targets for cancer treatment, which recognize DNA strands and impede cancer cell proliferation^7–9^.

Proper regulation of DNA replication is crucial for ensuring stable genome inheritance and cell division. Recent studies have profiled the protein expression of the DNA replication licensing factor, minichromosome maintenance protein 2–7 complex (MCM2–7), which is correlated with cancer progression^10^. During the initiation of DNA replication, the heterohexameric complex MCM2-7 unwinds double-stranded DNA and forms a replication fork^11^. The MCM2-7 limits DNA replication to a single occurrence per cell division, only binding onto DNA sequences with low levels of cyclin-dependent kinase (CDK) activity, along with an origin recognition complex (ORC), CDC6, and CDT1 during the G1 phase^12–14^. Despite efforts to comprehend how phosphorylation regulates initiation of DNA synthesis^15,16^, the biological networks of MCM2-7 in lung cancer cells via protein phosphorylation remain unmapped.

Protein phosphorylation is a post-translational modification that governs most of the signal transduction and regulates a variety of cellular processes, including the cell cycle, growth, apoptosis, and differentiation^17,18^. Controlled by kinases and phosphatase, protein phosphorylation is the most common reversible enzyme-catalyzed modification. The total number of modification sites is 338,948 on 20,368 proteins, where seventy-three percent are phosphorylation, 15% ubiquitination and 8% acetylation^19^. To date, a global analysis of serine-, threonine-, and tyrosine-phosphorylation has been performed using advanced mass-spectrometric techniques combined with hydroxy acid-modified metal oxide chromatography (HAMMOC)^20,21^. HAMMOC is a precise and popular phosphopeptide enrichment method that uses metal oxide chromatography modified with aliphatic hydroxyl acids to reduce the non-specific binding of acidic amino acid for large-scale study of phosphorylated proteins^22–24^.

The MCM proteins including MCM2-MCM7 were first identified in yeast and known to be the core of the replicative helicase for DNA replication^25,26^. Deregulation of MCM proteins have been reported as promising prognostic markers for lung cancer^27–32^; however, the role of MCM proteins in cancer formation is contradictory as both overexpression and reduction of MCM proteins are associate with cancer development^10^. The six distinct MCM proteins form into a ring-shaped complex to manipulate DNA within their central tunnel^33^. During DNA replication, the MCM complex cannot bind to double-stranded DNA in a ring shape, therefore, an opening at MCM2/5 interface is required for DNA loading^34^. The regulation of the MCM2/5 gate conformation inhibits DNA synthesis and activates the binding of MCM2-7 around DNA^35,36^. Phosphorylation of MCM2 occurs at multiple sites, which results in a conformational change in the complex and activation of helicase activity^37^. The protein phosphorylation response to MCM2 in lung cancer remains uncharacterized; however, MCM2 has been proposed as an excellent proliferation marker in many types of cancer^38–40^. In this study, we perform large-scale analysis of the phosphoproteome and proteome to characterize and interpret MCM2, in an attempt to establish a global functional distribution of the identified phosphoproteins and phosphosites in both overexpressed and silenced MCM2 lung cancer cells. Our results provide a comprehensive insight into the regulatory role of MCM2 in lung cancer, and also reveal that MCM2 promotes cell proliferation might possibly via the regulation of high mobility group protein HMG-I/HMG-Y (HMGA1) phosphorylation. Understanding the molecular interactions of MCM2 in lung cancer cells enhances our capacity to therapeutically target cancer-specific phosphoproteins.

## Results

### Overexpression of MCM2 correlates with poor survival rate in lung cancer patients

**Figure 1:**
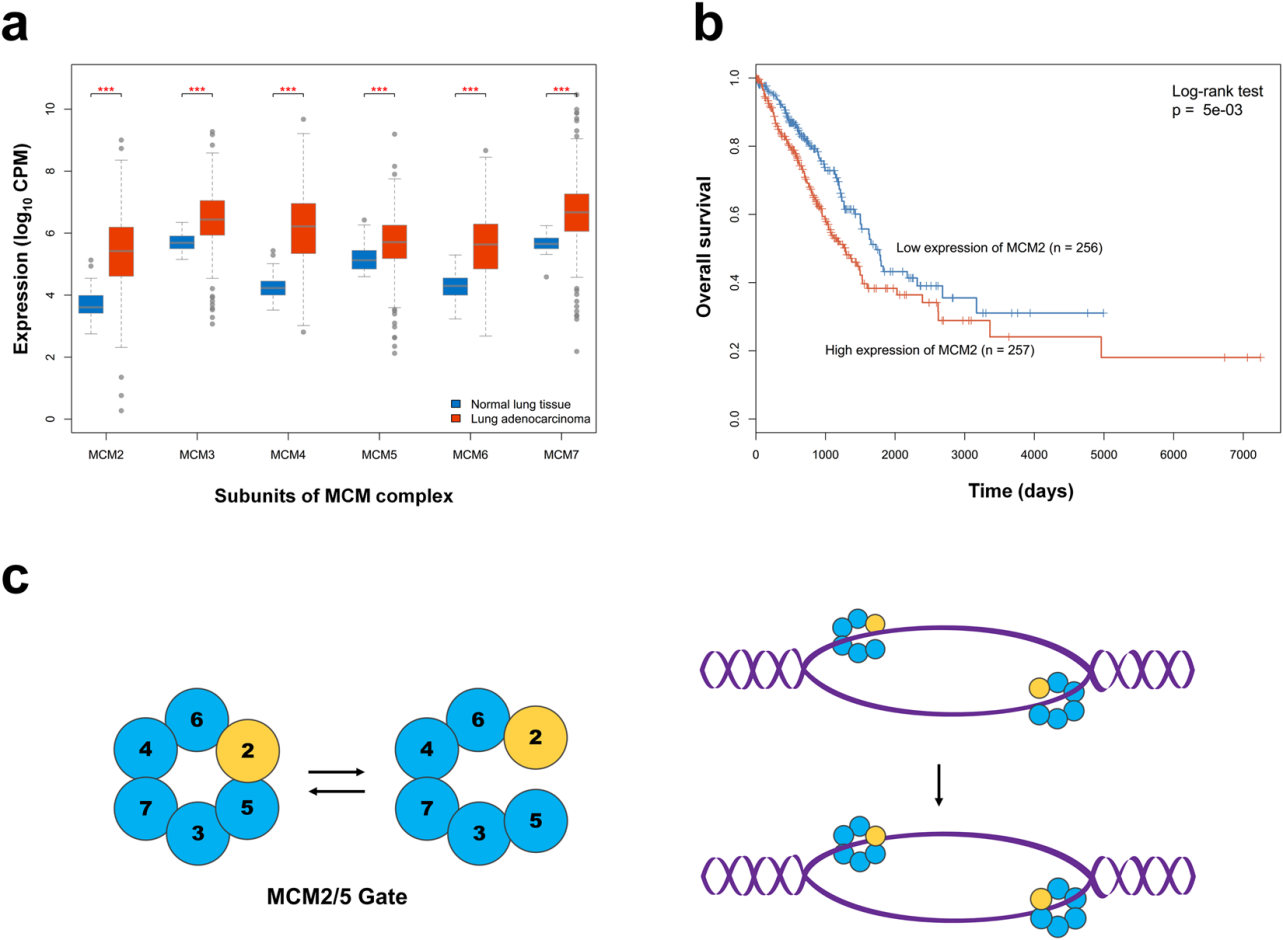
MCM gene expression in lung adenocarcinoma. (**a**) Expression levels of six MCM complex genes (MCM2-7) in normal lung tissue and lung adenocarcinoma. Each gene is represented by two mean values derived from its expression in 59 normal (blue) and 515 lung adenocarcinoma (red) samples. CPM: counts per million; ***p < 0.001. (**b**) Kaplan-Meier plots showing overall survival rates for lung adenocarcinoma in two groups separated according to levels of MCM2 expression: high (red) and low (blue). (**c**) Schematic presentation of the regulation of MCM2/5 gate conformation, which restrains DNA synthesis and activates the MCM2-7 complex to encircle the DNA.

To examine the clinical significance of MCM subunits expression in lung adenocarcinoma, we analyzed the RNA-seq dataset of lung adenocarcinoma from The Cancer Genome Atlas (TCGA) which contains 515 cancerous tissues and 59 adjacent normal tissues. All six MCM2-7 genes were significantly overexpressed in lung adenocarcinoma relative to normal lung tissues (Fig. 1a). Moreover, we stratified tumors based on the median of a given subunit expression, and found that the tumors with high MCM2 or MCM5 expression carry a poor prognosis (log-rank test p < 0.01) (Fig. 1b and Supplementary Fig. S1). Although MCM2 and MCM5 both play as the DNA entry gate of the MCM complex to regulate the initiation of DNA replication (Fig. 1c) and might be important in regulating lung cancer, we only focused on studying MCM2. We further examined MCM2 expression in 14 cancer types from TCGA, and found MCM2 is significantly highly expressed in all cancerous tissues in comparison to their adjacent normal tissues (Supplementary Fig S2).

**Figure 2:**
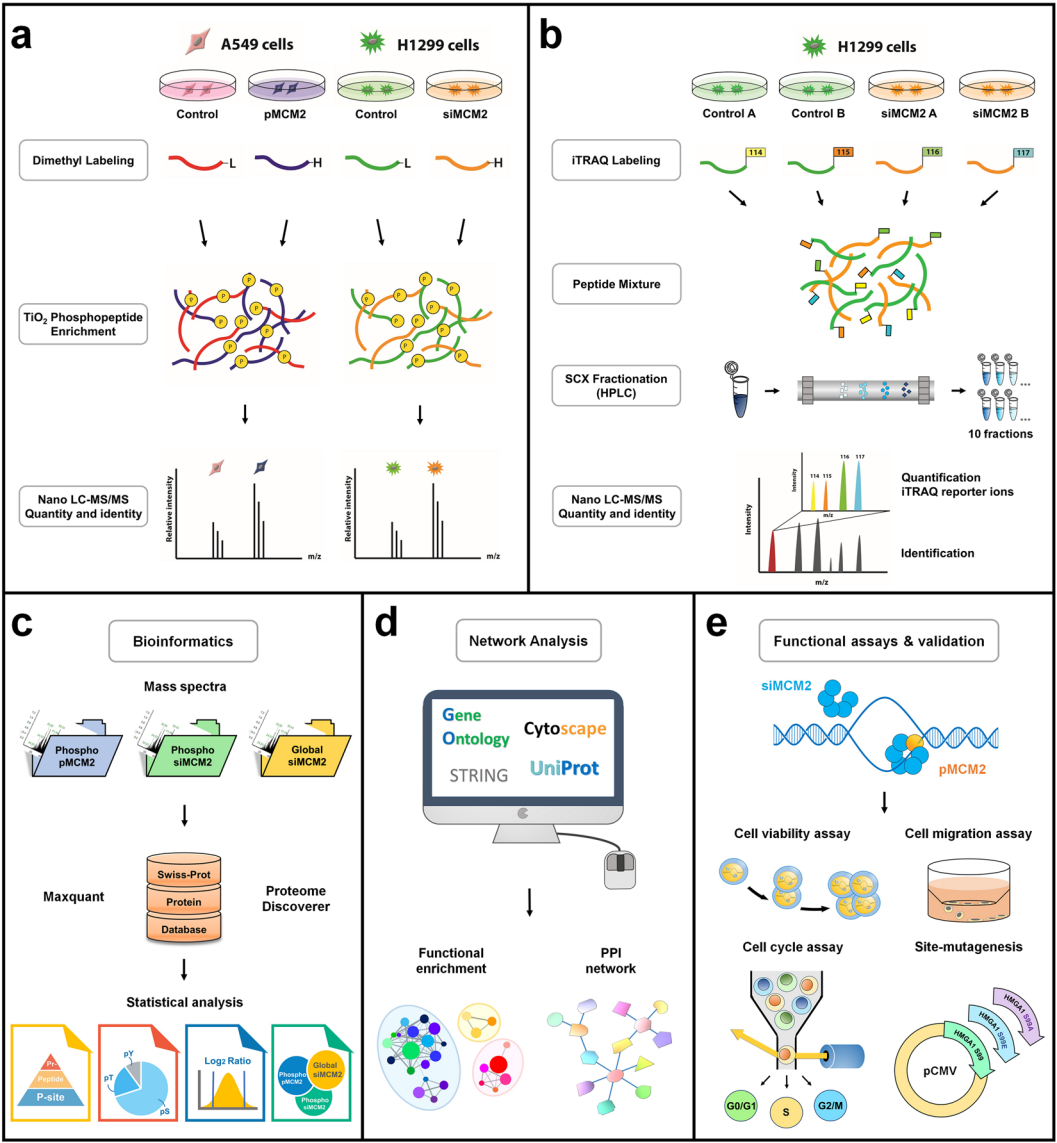
Overall workflow for integrated profiling of the phosphoproteome and global proteome regulated by MCM2 in non-small cell lung cancer cells. (**a**) Experimental strategy for quantitative phosphoproteomic profiling in response to overexpression of MCM2 (pMCM2) in A549 cells and silencing of MCM2 (siMCM2) in H1299 cells. Protein extracts obtained from the transfected cells were digested, dimethyl labeled, phosphopeptide enriched, and analyzed with mass spectrometry. (**b**) Experimental strategy for quantitative global proteomic profiling in response to siMCM2 in H1299 cells. Protein extracts obtained from the transfected cells were digested, iTRAQ labeled, SCX fractionated, and analyzed with mass spectrometry. (**c**) MCM2 phosphoproteomic and global proteomic mass spectra were identified and quantified using MaxQuant or Proteome Discoverer and analyzed using a bioinformatics strategy. (**d**) Construction of functional network and protein-protein interaction from differentially expressed phosphoproteins and proteins. (**e**) MCM2-perturbed biological processes in lung cancer cells were validated by functional assays, and the protein of interest was further investigated by site-directed mutagenesis.

In order to comprehensively analyze the role of MCM2 in lung cancer, two NSCLC cell lines, A549 and H1299 with different endogenous expression levels of MCM2 were used (Supplementary Fig. S3). Our experimental strategy aimed to identify MCM2-induced changes by silencing MCM2 in H1299 cells while overexpressing it in A549 cells (Supplementary Fig. S4). In this study, quantitative phosphoproteomic (Fig. 2a) and global proteomic profiles (Fig. 2b) were designed for the MCM2 response profile. Taken collectively, these observations provide an integrated analysis combining MS-based discovery, bioinformatics analyses (Fig. 2c and d), and selective functional assays (Fig. 2e) to generate hypothesis-driven targets for lung cancer drug development.

### Quantitative phosphoproteome of lung cancer cells regulated by MCM2

**Figure 3:**
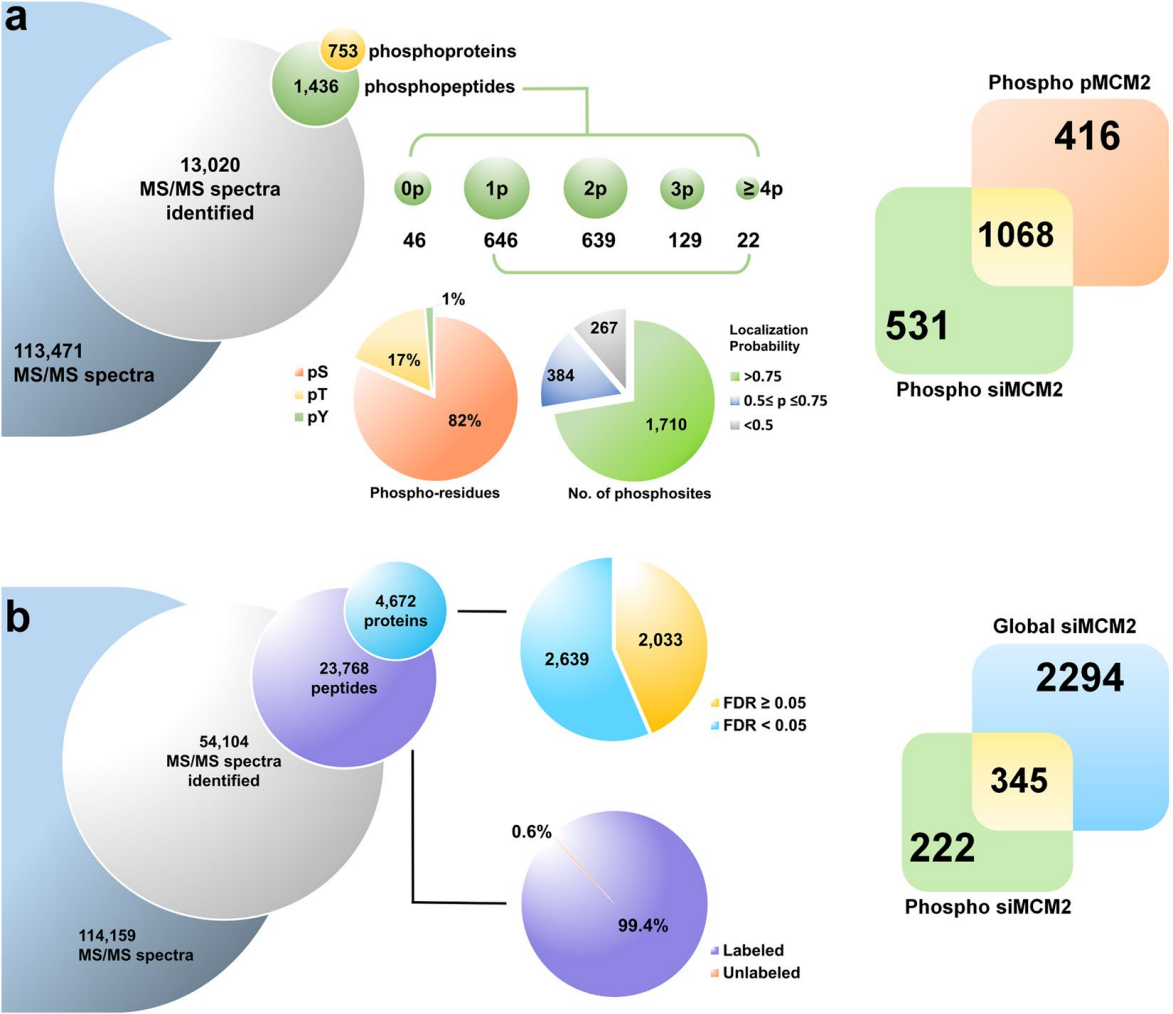
Quantitative phosphoproteome and global proteome profiling of MCM2 overexpression and silencing in lung cancer cells. (**a**) Quantitation and identification of the phosphoproteome of lung cancer cells in response to siMCM2 and pMCM2 in A549 and H1299 cells. Venn diagram illustrating the overlap in the sets of phosphoproteins. (**b**) Quantitation and identification of the global proteome of lung cancer cells in response to siMCM2 in H1299 cells. Venn diagram illustrating the overlap in the set of siMCM2 phosphoproteins with that of the siMCM2 global proteome.

To identify previously unmapped MCM2-responsive phosphorylation proteins and gain system-wide insights into the regulatory role of MCM2 in lung cancer cells, we performed a quantitative phosphoproteomic analysis on 24-h overexpressed-MCM2 (pMCM2) in A549 cells and 48-h silenced-MCM2 (siMCM2) in H1299 cells (Fig. 2a). The protein phosphorylation profile in response to MCM2 was dimethyl labeled and enriched by HAMMOC^23,41,42^ followed by nanoscale liquid chromatography-tandem MS (LC-MS/MS). We identified a total of 1436 phosphopeptides that mapped to 2361 phosphorylation sites on 753 phosphoproteins in MCM2-overexpressed A549 and MCM2-silenced H1299 cells (Fig. 3a, Supplementary Tables S1 and S2). The quantitative phosphoproteome mapping of MCM2 revealed that the majority of phosphopeptides were singly or doubly phosphorylated, yielding a Ser:Thr:Tyr phosphorylation ratio of 82:17:1 (Fig. 3a). Among the 2361 quantified phosphosites, 1710 were assigned with a high localization probability (p > 0.75) (Fig. 3a, Supplementary Fig. S5, and S6). To determine the phosphosites with significant phosphorylation change, we applied a threshold of the normalized H/L ratio <0.67 (1.5-fold reduced) or >1.5 (1.5-fold increased) and *p*-value < 0.05 derived from Significance B on these high confident phosphosites^43^. We identified 215 and 107 phosphosites that were significantly regulated in response to MCM2 overexpression and silencing respectively (Supplementary Tables S3 and S4).

### Quantitative proteome of lung cancer cells regulated by MCM2

In order to obtain a more comprehensive understanding of the regulatory networks of MCM2-modulated protein phosphorylation in lung cancer cells, the global proteome was investigated using iTRAQ labeling on 48-h silenced-MCM2 H1299 cells (Fig. 2b). Global profiling of the quantitative proteome was obtained from two small interfering RNA (siRNA) controls (iTRAQ-labeled 114 and 115) and two siMCM2 (iTRAQ-labeled 116 and 117) that were used to knock down MCM2 in H1299 cells. Ten SCX fractions were analyzed individually by LC-MS/MS to both identify and obtain quantitative information about the MCM2-perturbated proteomic profile. There were a total of 54104 MS/MS spectra, 23768 peptides, and 4672 proteins identified, with an iTRAQ labeling efficiency of 99.4% (Fig. 3b). The peptides that had been mapped from at least two unique iTRAQ-labeled peptides were used to identify proteins and quantify results with a high degree of confidence (Supplementary Table S5). The iTRAQ quantitative proteomic measurements revealed high reproducibility; the R^2^ of regression models between individual peptide signal replicates ranged from 0.990 to 0.996 (Supplementary Fig. S7). Using 1.5-fold changes as the threshold for significantly regulated proteins, we identified 46 differentially expressed proteins (Supplementary Fig S8 and Table S6).

### Functional networks of MCM2-regulated proteins

**Figure 4:**
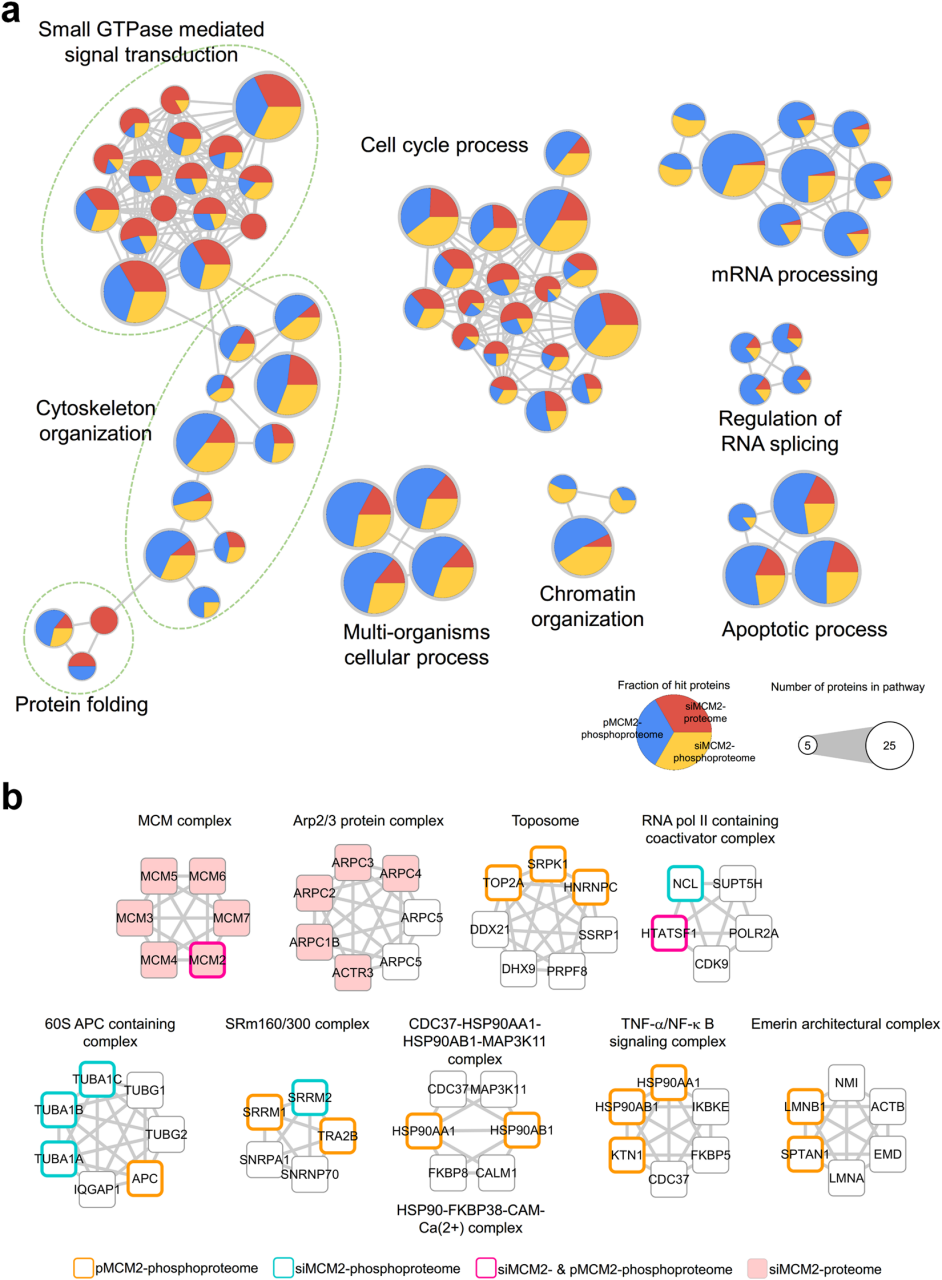
Functional networks of MCM2-regulated phosphoproteins. (**a**) Functional enrichment analysis of proteins with differential expression under MCM2 silencing (red) and proteins with differential change in phosphorylation levels under MCM2 overexpression (blue) or silencing (yellow). According to corrected *p*-value < 0.05, 76 over-represented GO terms were identified. (**b**) Ten protein complexes that are over-represented in response to MCM were obtained from CORUM. Variable protein members of the complex are colored depending on the phosphoproteomic or global proteomic profiles.

We performed Gene Ontology (GO) enrichment analyses of proteins that exhibited either differential expression under MCM2 silencing or differential change in phosphorylation levels under MCM2 overexpression or silencing. With a corrected *p*-value of <0.05, 76 over-represented GO terms were identified (Table S7) and graphically illustrated as an enrichment map (Fig. 4a). In the enrichment map, the enriched GO terms are grouped into the following functional clusters: the regulation of chromatin organization, RNA splicing, mRNA processing, cell cycle process, protein folding, small GTPase mediated signal transduction, and cytoskeleton organization (Fig. 4a).

Unsurprisingly, a group of proteins from the three MCM2-perturbated profiles is involved in aspects of DNA replication, including DNA unwinding, DNA strand elongation, and initiation (Supplementary Table S7). This is consistent with the central role of the MCM2 protein. Many biological processes related to RNA and mRNA processing and splicing are enriched from the differentially expressed proteins in response to MCM2. Aberrant RNA splicing is commonly linked to cancer-related functions in which defects in alternative splicing lead to various human diseases – primarily cancer progression^44,45^. Alternative splicing generates protein isoforms that can be involved in any aspect of tumor progression and maintenance and may promote cancer cell proliferation, migration, and invasion, and affect metabolism^46,47^.

Some proteins in response to the perturbation of MCM2 are associated with the process of cell cycle control (Fig. 4a and Supplementary Table S7). The transition from the G1 to S phase of the cell cycle is essential for the regulation of cell proliferation. During the G1 phase, cyclin-dependent kinase (CDK) activity promotes DNA replication and initiation of the progression to the S phase, and its deregulation promotes cancer progression^48^. In addition, many cell-cycle-related biological processes are also enriched such as the regulation of DNA conformational changes, chromosome and chromatin organization, and initiation of DNA replication (Supplementary Table S7).

We also identified 10 over-represented protein complexes from CORUM (Fig. 4b and Supplementary Table S8). Some of these protein complexes are related to actin and cytoskeleton organization, including Arp2/3 and emerin architectural complexes. These findings suggest that the deregulation of MCM2 might be involved in cell proliferation, migration and the cell cycle.

### Validation of MCM2 functional networks on cell proliferation, cell cycles, and migration in lung cancer cells

**Figure 5:**
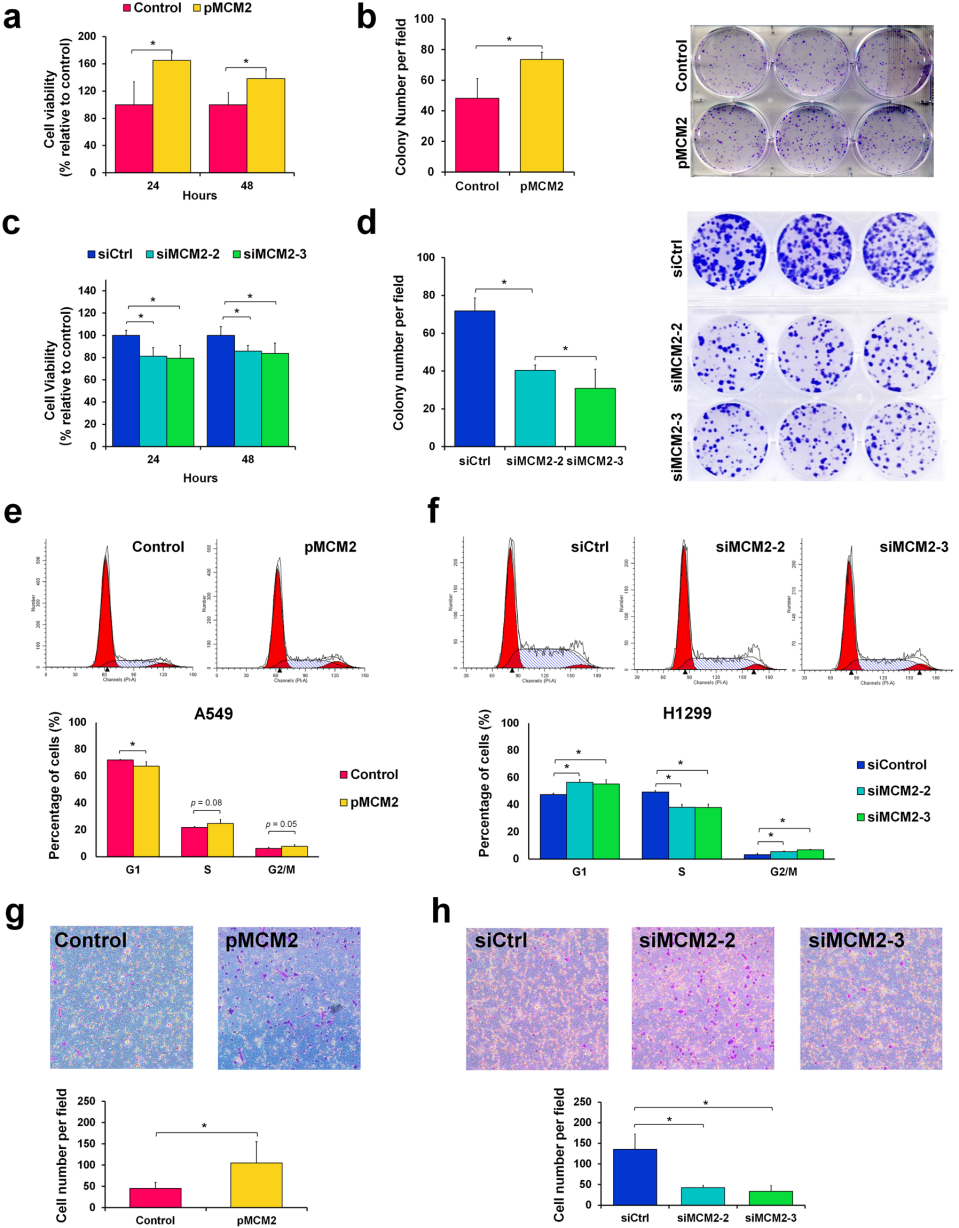
MCM2 regulates cell proliferation, the cell cycle via G1/S phase arrest, and cell migration in lung cancer cells. (**a**) Overexpression of MCM2 (pMCM2) enhanced cell proliferation at 24 h and 48 h post-transfection, as assessed by MTT assay. (**b**) The effects of MCM2 overexpression on colony formation in A549 cells transfected with MCM2, compared with the control. MCM2-overexpressing A549 cells exhibited a significant (53%) increase in colony formation activity. (**c**) Silencing of MCM2 (siMCM2-2 and siMCM2-3) repressed cell proliferation at 24 h and 48 h post-transfection, as assessed by MTS assay. (**d**) MCM2-silenced H1299 cells exhibited a significant decrease in colony formation activity: 44% (siMCM2-2) and 57% (siMCM2-3). (**e**) Overexpression of MCM2 in A549 cells interrupt the cell cycle process in the G1 phase. A549 cells were transfected with pMCM2 or pcDNA3.1(+) control for DNA content analysis using FASC. The abundance of MCM2-overexpressed A549 cells in the G1 phase decreased and that of cells in the S phase and G2/M phase slightly increased. (**f**) Silencing of MCM2 in H1299 cells induced cell cycle arrest at the G1/S phase. H1299 cells were transfected with MCM2-siRNA (siMCM2-2 and siMCM2-3) or control-siRNA. The abundance of siMCM2-silenced H1299 cells in the G1 phase increased and that of cells in the S phase decreased. An accumulation of cells in the G2/M phase was also observed in MCM2-silenced cells. (**g**) Overexpression of MCM2 promotes cell migration, as shown by Transwell migration assays. Microscopic image of crystal violet staining and bar plot showing that A549 cells transfected with pMCM2 had a greater migratory ability than the pcDNA3.1(+) control. (**g**) Silencing of MCM2 in the H1299 cell line represses cell migration. Microscopic image of crystal violet staining and bar plot showing that H1299 cells transfected with MCM2-siRNA had a lower migratory ability than the siRNA control. *p < 0.05.

To investigate the effects of MCM2 in lung cancer, cell viability and colony formation assays in response to MCM2 expression manipulation was performed (Fig. 5a–d). Cell proliferation was strongly affected by MCM2; the viability of MCM2-overexpressing (pMCM2) A549 cells increased significantly by 65.0% and 38.2%, at 24 h and 48 h, respectively (Fig. 5a). Colony formation was monitored to investigate the long-term effect of MCM2 on lung cancer cell proliferation, and the results indicated that overexpression of MCM2 improved colony-forming ability (Fig. 5b). To ascertain the stimulatory effect of MCM2 on lung cancer cells, we used small interfering RNA (siRNA) to knock down MCM2 in H1299 cells, which caused a noticeable decline in cell proliferation. The cell viability of MCM2-silenced H1299 cells (siMCM2-2 and siMCM2-3) also decreased significantly, by 18.8% and 20.7% at 24 h, and by 14.2% and 16.3% at 48 h, respectively (Fig. 5c). The long-term effect of MCM2 on H1299 cell indicated that silencing of MCM2 also inhibited colony forming ability (Fig. 5d). These observations suggest that MCM2 is required for optimal cell proliferation and serves a regulatory purpose in lung cancer cell proliferation.

Since the differentially regulated proteins were principally involved in the cell cycle from the functional network analysis, we hypothesized that there would be an attendant perturbation in cell cycle regulation. Flow cytometry for DNA content was conducted to evaluate whether MCM2 perturbation can interrupt cell cycle progression in A549 and H1299 cells (Fig. 5e and f). The distribution of cells in the different phases of the cell cycle was analyzed, and the results showed that the percentage of G1 phase in MCM2-overexpressed A549 cells decreased by 4.77%, and that of the S phase and G2/M phase slightly increased by 3.08% and 1.69% relative to pcDNA3.1(+) A549 control cells, respectively (Fig. 5e). Contrastingly, flow cytometry revealed that the percentage of cells in the G1 phase of MCM2-silenced (siMCM2-2 and siMCM2-3) H1299 cells increased by 8.43%, and that of the S phase decreased by approximately 11.39% relative to siRNA control H1299 cells (Fig. 5f), indicating a G1/S arrest and inhibition of DNA synthesis. The accumulation of cells in the G2/M phase, however, increased by 2.97% in MCM2-silenced cells relative to siRNA control cells (Fig. 5f). These results indicate that the perturbation of MCM2 in lung cancer cells causes cell cycle interruption at the transition between the G1 and S phases.

Based on the results of GO enrichment analysis (Fig. 4a), both the overexpression and silencing of MCM2 led to the enrichment of genes involved in the regulation of cytoskeleton organization, actin filament polymerization, and microtubule-based movement (Supplementary Table S7), and we suggest that MCM2 has a close connection to cell migration ability. To explore the role of MCM2 in cell motility and migration, we conducted a Transwell migration assay to examine the migration ability of MCM2-overexpressing and MCM2-silenced cells at 48 h post-transfection. We observed that the cell migration ability of MCM2-overexpressing cells increased significantly, by 132% (Fig. 5g), while that of MCM2-silenced cells decreased significantly (by 68.6% and 75.4%) (Fig. 5h). These results further support the notion that MCM2 plays an important role in the cell migration of lung cancer cells.

### Identification of the MCM2-associated phosphoproteins

**Figure 6:**
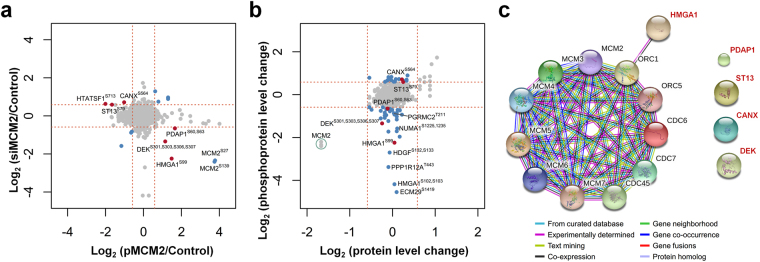
Identification of MCM2-associated phosphoprotein HMGA1. (**a**) Comparison of phosphoprotein expression ratios with specific phosphorylation sites from the MCM2 overexpression and silencing phosphoproteomic profiles for two lung cancer cell lines. Phosphoproteins with specific sites that were significantly up- or down- regulated, with at least a 1.5-fold change in opposite directions in the pMCM2 and siMCM2 phosphoproteomes are represented in red. (**b**) Comparison of changes in protein abundance from the global siMCM2 proteome with changes in phosphoprotein expression ratios from the siMCM2 phosphoproteome in lung cancer cells. Phosphoproteins that were significantly up- or down- regulated by siMCM2 but with no change in protein abundance from the proteome are represented in blue. The phosphoproteins with specific phosphorylation sites that changed in opposite directions in the pMCM2 and siMCM2 phosphoproteomic profiles, without changes in protein abundance, are represented in red. (**c**) The network predicted a functional association between HMGA1 and the MCM complex. Five MCM2-associated phosphoproteins (HMGA1, PDAP1, DEK, ST13, and CANX) and components of MCM complex are used as seeds to construct a functional association network via STRING.

Since overexpression and silencing of MCM2 are two contrasting perturbations, our analysis focused only on those phosphoproteins exhibiting differential abundance in the two phosphoproteome profiles analyzed. These phosphoproteins should represent those sites on the candidate proteins that have the highest probability of being regulated by MCM2. A total of 1068 overlapping phosphosites were identified from both overexpressed-MCM2 (pMCM2) and silenced-MCM2 (siMCM2) phosphoproteome profiling (Fig. 3a). Six proteins with 10 specific phosphosites were found to be differentially regulated by both pMCM2 and siMCM2, with opposing phosphorylation patterns (Fig. 6a). HIV Tat-specific factor (HTATSF1), Calnexin (CANX), and Hsc70-interacting protein (ST13) were down-regulated in pMCM2 and up-regulated in siMCM2 in both A549 and H1299 cells. Similarly, 28 kDa heat- and acid-stable phosphoprotein (PDAP1), High mobility group protein HMG-I/HMG-Y (HMGA1), and Protein DEK (DEK) were found to be significantly regulated by siMCM2 and pMCM2 in opposite ways.

We next compared the siMCM2 phosphoproteomic profile with the siMCM2 proteomic profile to identify MCM2 interactions that merit the effort and expense of full validation. There were 345 proteins identified from both the siMCM2 phosphoproteome and proteome profiles (Fig. 3b), most differentially expressed phosphoproteins have no change in abundance from proteomic profile (Table 1), only six of which differentially expressed phosphoproteins that were found in the proteomic profile, including MCM2 itself, suggesting that these differentially expressed phosphoproteins were regulated by phosphorylation. Furthermore, a scatter plot of siMCM2 phosphosites and siMCM2 global proteins reveals five out of the six MCM2-associated phosphoproteins (HMGA1, PDAP1, DEK, ST13, and CANX) did not change in abundance from the global proteomic profile (Fig. 6b).

**Table 1:** Significantly regulated phosphosites in siMCM2 phosphoproteome with no change in protein abundance from the global siMCM2 proteome in H1299 lung cancer cells.

**Protein**	**Proteome Log**_**2**_ **ratio (siMCM2/CTL)** ^**a**^	**Phosphoproteome Log**_**2**_ **ratio (siMCM2/CTL)** ^**b**^	**Phosphorylation sites on protein** ^**c**^	**Peptide sequence** ^**d**^	**Phosphorylation sites on peptide** ^**e**^
BOD1L1	0.09	0.73	Ser^2779; 2780^	_QHYL**pSpS**EDEPDDNPDVLDSR_	5; 6
BOP1	−0.31	−0.59	Ser^126; 127^	_IGDEYAED**pSpS**DEEDIR_	9; 10
CANX	0.22	0.71	Ser^564^	_QKSDAEEDGGTV**pS**QEEEDR_	13
CANX	0.22	−0.94	Thr^562^	_QKSDAEEDGG**pT**VSQEEEDR_	11
CCDC132	−0.09	−0.67	Ser^559^	_SAYQEYD**pS**DSDVPEELK_	8
CCDC86	−0.21	−1.08	Ser^69^	_AGLGSPERPPKTSPG**pS**PR_	16
CCDC86	−0.21	−0.98	Ser^110^	_QQDLHLESPQRQPEY**pS**PESPR_	16
CCDC86	−0.21	−0.84	Thr^65^	_AGLGSPERPPK**pT**SPGSPR_	12
CLIP1	0	0.83	Ser^200; 204^	_TASESISNL**pS**EAG**pS**IK_	10; 14
DBN1	0.33	0.9	Ser^141^	_L**pS**SPVLHR_	2
DEK	−0.23	−1.34	Ser^301; 303; 306; 307^	_KE**pS**E**pS**ED**pSpS**DDEPLIKK_	3; 5; 8; 9
ECM29	0.05	-4.55	Ser^1419^	_**pS**CAFAMGHLVRTSRDSSTEK_	1
HDGF	−0.07	−2.7	Ser^132; 133^	_KGNAEG**pSpS**DEEGKLVIDEPAK_	7; 8
HIST1H1B	−0.04	−1.23	Ser^18^	_SETAPAETATPAPVEK**pS**PAK_	17
HMGA1	0.05	−2.24	Ser^99^	_KLEKEEEEGI**pS**QESSEEEQ_	11
HMGA1	0.05	−4.19	Ser^102; 103^	_KLEKEEEEGISQE**pSpS**EEEQ_	14; 15
HNRNPUL2	−0.08	0.7	Ser^161^	_REEDEPEER**pS**GDETPGSEVPGDK_	10
HNRNPUL2	−0.08	0.82	Thr^165^	_REEDEPEERSGDE**pT**PGSEVPGDK_	14
MYBBP1A	0.11	−1.96	Ser^1159^; Thr^1161^	_EIP**pS**A**pT**QSPISK_	4; 6
MYH9	0.01	−0.87	Ser^1943^	_GAGDG**pS**DEEVDGKADGAEAKPAE_	6
NCL	0.03	−0.93	Ser^563^	_LELQGPRG**pS**PNAR_	9
NOC2L	0.04	−0.73	Ser^49^	_EAAR**pS**PDKPGGSPSASR_	5
NOC2L	0.04	−0.74	Ser^56^	_EAARSPDKPGG**pS**PSASR_	12
NUMA1	0.07	−1.58	Ser^1225; 1235^	_N**pS**LISSLEEEV**pS**ILNR_	2; 12
PA2G4	−0.02	0.68	Thr^386^	_TAENATSGE**pT**LEENEAGD_	10
PDAP1	−0.06	−0.65	Ser^60; 63^	_SLD**pS**DE**pS**EDEEDDYQQK_	4; 7
PGRMC2	0.04	−0.88	Thr^211^	_LLKPGEEPSEY**pT**DEEDTK_	12
PLEC	−0.08	−0.78	Ser^1435^	_AQLEPVA**pS**PAK_	8
PPP1R12A	−0.16	−0.67	Ser^862^	_STGVSFWTQDpSDENEQEQQ**pS**DTEEGSNKK_	11
PPP1R12A	−0.16	−1.73	Ser^445^	_TG**pS**YGALAEITASK_	3
PPP1R12A	−0.16	−3.38	Thr^443^	_**pT**GSYGALAEITASK_	1
RANBP2	0.11	0.72	Thr^1393^; Ser^1400^	_ELVGPPLAETVF**pT**PKT**pS**PENVQDR_	10; 17
RBM14	−0.19	0.66	Ser^582^	_TRL**pS**PPR_	4
RPS6	0.03	−0.71	Ser^235^	_RL**pS**SLRASTSK_	3
RRM2	−0.42	−0.71	Ser^20^	_VPLAPITDPQQLQL**pS**PLK_	15
RRP1B	0.00	0.61	Ser^245^	_VGDGDL**pS**AEEIPENEVSLR_	7
RTN4	0.26	0.7	Ser^184^	_RGSSG**pS**VDETLFALPAASEPVIR_	6
SEC. 31 A	−0.1	0.6	Ser^532^	_DSDQVAQSDGEE**pS**PAAEEQLLGEHIK_	13
SPTBN1	0.26	0.65	Ser^2164^	_E**pS**SPIPSPTSDRK_	2
SPTBN1	0.26	0.7	Ser^2165^	_TSSKES**pS**PIPSPTSDRK_	7
SRRM2	0.06	−1.16	Ser^351^	_SATRP**pS**PSPERSSTGPEPPAPTPLLAER_	6
SRRM2	0.06	−0.86	Ser^353^	_SATRPSP**pS**PERSSTGPEPPAPTPLLAER_	8
SRRM2	0.06	−1.02	Ser^357^	_SATRPSPSPER**pS**STGPEPPAPTPLLAER_	12
SRRM2	0.06	−1.71	Ser^2692; 2694^	_SL**pS**Y**pS**PVER_	3; 5
SRSF1	−0.18	−0.96	Ser^201; 205^	_VDGPRSP**pS**YGR**pS**R_	8; 12
ST13	0.23	0.59	Ser^79^	_KVEEDLKADEPSSEE**pS**DLEIDK_	16
STUB1	−0.17	−0.69	Ser^19; 23^	_LGAGGG**pS**PEK**pS**PSAQELK_	7; 11
THRAP3	−0.19	0.6	Ser^379^	_GSF**pS**DTGLGDGK_	4
TNKS1BP1	0.23	0.64	Ser^1666^	_NR**pS**AEEGELAESK_	3
TUBA1C	0.18	−0.71	Ser^48^	_TIGGGDD**pS**FNTFFSETGAGK_	8
UTP18	−0.33	−1.11	Ser^205^	_RKT**pS**SDDESEEDEDDLLQR_	4
UTP18	−0.33	−0.76	Ser^210^	_TSSDDE**pS**EEDEDDLLQR_	7

^a^Log_2_ value of the normalized siMCM2/siRNA control ratio from siMCM2 proteome in H1299 lung cancer cells.

^b^Log_2_ value of the normalized siMCM2/siRNA control ratio from siMCM2 phosphoproteome in H1299 lung cancer cells.

^c^Position(s) of the phosphorylated residue within this protein.

^d^Sequence of matched peptide including the location(s) of phosphorylated amino acid.

^e^Position(s) of the phosphorylated residues within this peptide.

To investigate the functional associations between MCM2 and MCM2-associated phosphoproteins, i.e. HMGA1, PDAP1, DEK, ST13, and CANX, we constructed a functional association network via STRING^49^. The MCM2 and MCM2-associated phosphoproteins were used as seeds, and to obtain more associations among seed proteins, we set the STRING interaction confidence as 0.36. Interestingly, only HMGA1 has a functional association with MCM complex, through an ORC complex (Fig. 6c). Indeed, many studies have shown that HMGA1 interacts with ORC complex to regulate the replication origins^50–52^. However, the relationship between HMGA1 and MCM2 is still unclear.

### MCM2 regulates HMGA1 Ser99 in determining lung cancer cell viability

**Figure 7:**
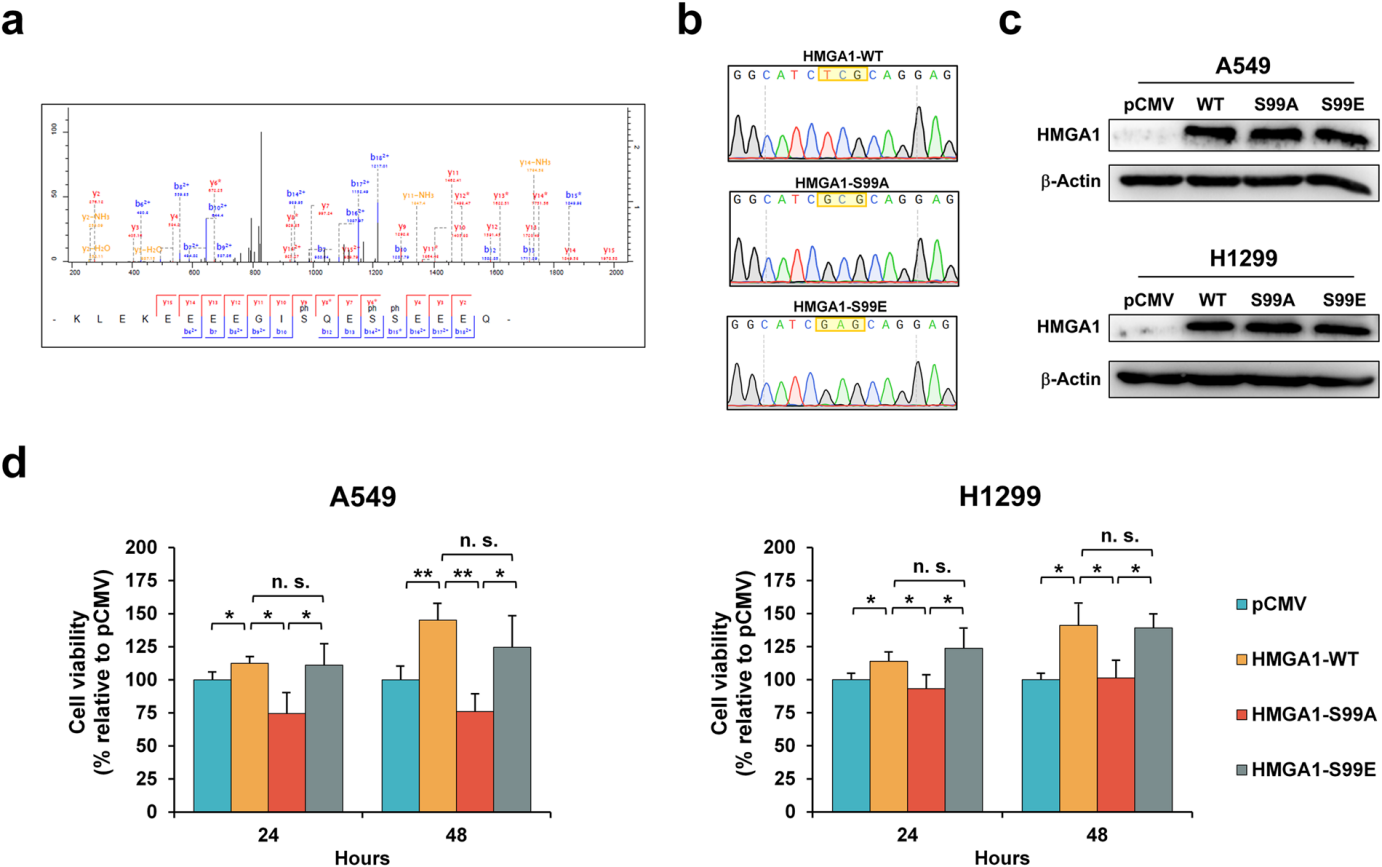
Phosphorylation of HMGA1 at Ser^99^ is involved in lung cancer cell proliferation. (**a**) Fragmentation spectrum for modified HMGA1 (High Mobility Group Protein HMG-I/HMG-Y) peptide, showing the phosphorylated serine residue 99. (**b**) Electropherogram representation of genomic DNA fragments from HMGA1^S99^ wild-type (top), the HMGA1^S99A^ mutant (middle) and HMGA1^S99E^ mutant (bottom), the positions of the mutations are indicated by yellow square. (**c**) Western blot showing the total HMGA1 protein expression of A549 and H1299 cells 48 h after transfection of pCMV vector (pCMV), HMGA1 wild-type (WT), HMGA1 ^S99A^ mutants (S99A), and HMGA1^S99E^ mutants (S99E). (**d**) Proliferation of A549 cells and H1299 under the overexpression of proteins HMGA1 wild-type (WT), HMGA1^S99A^ mutants (S99A), and HMGA1^S99E^ mutants (S99E), as assessed by MTS. Overexpression of HMGA1 wild-type and HMGA1^S99A^ mutants promoted A549 and H1299 cell proliferation at 24 h and 48 h post-transfection, whereas the non-phosphorylatable HMGA1^S99A^ mutant repressed A549 and H1299 cell proliferation at 24 h and 48 h post-transfection (*p < 0.05; **p < 0.01).

Based on these results, a common phosphorylation residue, Ser^99^, was identified in high mobility group protein HMG-I/HMG-Y (HMGA1) with a localization probability of 1.00, within both the overexpressed- and silenced-MCM2 datasets in opposite way (Fig. 7a and Table 2). Constitutive and inducible phosphorylation at the serine residues Ser^99^, Ser^101^, and Ser^102^ has been reported to be dependent on casein kinase 2 (CK2) specifically for DNA binding affinity^53–56^. In our phosphoproteome of MCM2 overexpression, tri-phosphorylated HMGA1 protein (Ser^99^, Ser^102^, and Ser^103^) was detected in MCM2-overexpressing A549 cells (Supplementary Table S9). On the other hand, the phosphoproteome of MCM2 silencing exhibited a significant decrease in the tri-phosphorylated HMGA1 protein at Ser^99^, Ser^102^, and Ser^103^ in the H1299 cells (Supplementary Table S10). Only Ser^99^ on HMGA1 was found to be differentially upregulated under MCM2 overexpression and differentially downregulated under MCM2 silencing, without any change in protein abundance in the proteomic profile, making this a reliable candidate for protein phosphorylation in response to MCM2 (Fig. 6a and b).

**Table 2:** Significantly regulated phosphosites in MCM2 phosphoproteome with no change in protein abundance from the global siMCM2 proteome in lung cancer cells.

Protein	Proteome	Phosphoproteome	Log_2_ ratio (pMCM2/CTL)^c^	Phosphorylation sites on protein^d^	Peptide sequence^e^	Phosphorylation sites on peptide^f^
	Log_2_ ratio (siMCM2/CTL)^a^	Log_2_ ratio (siMCM2/CTL)^b^				
CANX	0.22	**0.71**	**−1.02**	Ser^564^	_QKSDAEEDGGTV**pS**QEEEDR_	13
DEK	−0.23	**−1.34**	**1.15**	Ser^301; 303, 306; 307^	_KE**pS**E**pS**ED**pSpS**DDEPLIKK_	3; 5; 8; 9
HMGA1	0.05	**−2.24**	**1.48**	Ser^99^	_KLEKEEEEGI**pS**QESSEEEQ_	11
PDAP1	−0.06	**−0.65**	**1.65**	Ser^60; 63^	_SLD**pS**DE**pS**EDEEDDYQQK_	4; 7
PGRMC2	0.04	**−0.88**	**−0.65**	Thr^211^	_LLKPGEEPSEY**pT**DEEDTK_	12
NUMA1	0.07	**−1.58**	**−1.17**	Ser^1225; 1235^	_N**pS**LISSLEEEV**pS**ILNR_	2; 12
ST13	0.23	**0.59**	**−1.664**	Ser^79^	_KVEEDLKADEPSSEE**pS**DLEIDK_	16

^a^Log_2_ value of the normalized siMCM2/siRNA control ratio from siMCM2 proteome in H1299 lung cancer cells.

^b^Log_2_ value of the normalized siMCM2/siRNA control ratio from siMCM2 phosphoproteome in H1299 lung cancer cells.

^c^Log_2_ value of the normalized pMCM2/pcDNA 3.1(+) control ratio from pMCM2 phosphoproteome in A549 lung cancer cells.

^d^Position(s) of the phosphorylated residue within this protein.

^e^Sequence of matched peptide including the location(s) of phosphorylated amino acid.

^f^Position(s) of the phosphorylated residues within this peptide.

To further investigate the biological significance of HMGA1 phosphorylation at the serine residue at position 99 (Fig. 7a), we examined the cell viability of HMGA1 phosphorylation site mutant expressed in both A549 and H1299 cells. We constructed a recombinant wild-type of HMGA1, a non-phosphorylatable substitution with alanine, HMGA1^S99A^, and a phosphomimetic substitution with glutamic acid, HMGA1^S99E^ (Fig. 7b), where western blot analysis indicated that the transfection efficiencies were similar (Fig. 7c and Supplementary Fig. S9). Our results showed that overexpression of the HMGA1 wild-type and phosphomimetic HMGA1^S99E^ enhanced cell proliferation at 24 h and 48 h, whereas the mutation with a serine-to-alanine substitution (a dephosphorylated form of HMGA1^S99A^) resulted in decreased cell proliferation rates at 24 h and 48 h post-transfection suggesting that phosphorylation of HMGA1^S99^ contributes to cell proliferation in both A549 and H1299 lung cancer cells (Fig. 7d). These results suggest that the phosphorylation of HMGA1^S99^ seems to be a downstream phosphorylation event of MCM2 that plays an important role in the cell proliferation of lung cancer cells.

## Discussion

There are several treatments targeting MCM2 in colon and lung cancer, such as Trichostatin A and Lovastatin^57,58^; however, the molecular regulation in response to MCM2 via protein phosphorylation in lung cancer has not been fully elucidated. Additional study of MCM2 networks in lung cancer cells might expand our knowledge regarding lung cancer drug development and provide additional targets. Phosphorylation plays an essential role in normal physiological states, as well as in aberrant signaling pathways in cancer^59,60^. The MCM2-7 complex requires physical association of the accessory kinases CDC45 with the Go, Ichi, Nii and San (GINS) complex to unwind double-stranded DNA in an ATP-dependent reaction^61,62^. Our study has established a global functional distribution of the identified phosphoproteins and the phosphorylation sites involved in both the overexpression and the silencing of MCM2 in lung cancer cells. Integrating our analyses of the phosphoproteome and global proteome for two lung cancer cell lines in response to MCM2 provides a more comprehensive description of MCM2 downstream events and has allowed us to detect potential targets for drug development.

By combining statistical analysis with other computational tools, we were able to further characterize and evaluate a large number of phosphoprotein candidates. In this study, we pooled three sets of proteins with patterns of expression that differed in multiple ways, which can provide an understanding of the biological processes that are regulated by MCM2, by means of bioinformatics analysis. Based on this functional annotation, we performed cell viability assays in response to MCM2 and found that overexpression of MCM2 promotes cell proliferation in A549 cells, and MCM2 silencing inhibits cell proliferation in H1299 cells. Our results were consistent with the essential role of MCM proteins in cancer cell proliferation and tumorigenesis^63^. Previous studies have demonstrated that MCM2 knockdown leads to cell cycle arrest in colon and lung cancer cells^57,58^. Our observations that MCM2 knockdown deregulated the G1/S transition in lung cancer cells confirmed our hypothesis that MCM2 regulates the initiation of DNA replication, leading to cell proliferation. Further evidence of the role of MCM2 in regulating cell migration was obtained by using a Transwell assay to evaluate migration ability in MCM2-overexpressing A549 cells and MCM2-silenced H1299 cells, which showed that MCM2 knockdown inhibits cell migration in lung cancer cells. Our functional annotation analysis thus presents a reliable and accurate prediction that is consistent with the results of functional assays and merits full validation.

A combination of two contrasting perturbations—overexpression and silencing of MCM2—and an assessment of global protein abundance revealed the utility of our multi-dimensional proteomic approach. Five MCM2-associated phosphoproteins were identified from the three sets of profiles investigated. The protein association analysis revealed that only HMGA1 interacts with the MCM2-7 complex, pre-replication complex (ORC), and regulators of DNA replication and the cell cycle (CDC6, CDC7, and CDC45). The HMGA1 protein, as one of the HMG proteins (the most abundant non-histone chromatin-associated proteins), contains a unique DNA-binding domain that binds to the minor groove of AT-rich DNA sequences, and the binding modulates chromatins to regulate DNA-dependent processes such as replication and repair^64^. A recent study reported that elevated expression of HMGA1 correlates with malignant status and poor prognosis in NSCLC^65^. HMGA1 protein is strongly regulated by post-translational modifications, such as acetylation, methylation, and phosphorylation^56^. These findings indicate that phosphorylation of HMGA1 proteins is correlated with many cellular processes, including transcriptional regulation, the cell cycle, cell signaling, and apoptosis^66^. However, the association between the phosphorylation of HMGA1 and pre-initiation complex has not been thoroughly investigated. Our study found that phosphorylation of Serine 99 of HMGA1 is responsible for cell proliferation in lung cancer, implying that HMGA1 phosphorylation increases the DNA-binding activity of HMGA1, whereas dephosphorylation reduces its DNA-binding affinity, resulted in gene activation or repression, respectively.

In summary, our quantitative study of the phosphoproteome and proteome provides a comprehensive examination and validation of the effects of MCM2 protein expression in two lung cancer cell lines with opposing perturbations. Our investigation revealed the downstream events of phosphorylation that affect cancer cell proliferation, such as MCM2 promoting cell proliferation might possibly via the regulation of HMGA1 phosphorylation. Multi-dimensional proteomic approaches can also be applied to other diseases, since single proteomes cannot provide an unbiased interpretation of a disease.

## Materials and Methods

### Analysis of TCGA data set

Gene expression data of TCGA projects were downloaded from NCI Genomic Data Commons (https://gdc-portal.nci.nih.gov/). The read count dataset was normalized by the upper-quartile normalization and the expression of each gene was presented by log2-tranformated counts per million (log-CPM). In survival data analysis, Kaplan Meier estimator of survival rate was used to construct the survival curve, and log-rank test were used to compare overall survival between patients in different groups.

### Cell lines and cell culture

Human lung epithelial cells A549 (ATCC, CCL-185) and human non-small cell lung cancer cell HCI-H1299 (ATCC, CRL-5803) were obtained from the American Type Culture Collection (Manassas, VA, USA). The A549 cells were maintained in Dulbecco’s modified Eagle’s medium (catalog no. 12800-017; Gibco), and the H1299 cells were maintained in RPMI 1640 medium (catalog no. 31800-022; Gibco). All media was supplemented with 10% fetal bovine serum (catalog no. 04-001-1 A; Biological Industries) and grown at 37 °C in a humidified cabinet under 5% CO2.

### Plasmid construction and DNA manipulation

Total RNA was isolated by using TRIzol Reagent (Invitrogen) and subjected to DNase treatment (TURBO DNA-free; Ambion). PCR was performed to generate pcDNA3.1(+) plasmids (Invitrogen) with a full length sequence of MCM2. The MCM2 gene was amplified from synthesized cDNA (RevertAid First Strand cDNA synthesis kit; Thermo Fisher Scientific) by using the primer pair 5′-GCTAGCGCCACCATGGCGGAATCATCGGAA-3′, and 5′-CGCACGCGTACAAGCTTTCAGAACTGCTGCAGGAT-3′. The amplified DNA fragment was digested with restriction enzymes *Nhe*I and *Hind*III (New England Biolabs). The resulting plasmid (pcDNA3.1(+)-MCM2) was transformed into *Escherichia coli* (*E. coli*) strain DH5α and selected by antibiotics ampicillin. The plasmid DNA (pcDNA3.1(+)-MCM2) was prepared and sequenced at the DNA Sequencing Facility (Genomics BioSci. & Tech.). 2 × 10^5^ A549 cells were seeded 24 h before transfection in a 6-well plate using Lipofectamine 3000 (Invitrogen). Transiently transfected cells were harvested at 24 or 48 h post-transfection for further assays.

### RNA interference

Three commercial siRNA molecules against MCM2 (catalog no. SR302835; Origene) were transfected into H1299 cells to generate the transient silencing of MCM2 using Lipofectamine 3000 (Invitrogen). Five thousand H1299 cells were seeded on six-well plates 24 h before transfection. Cells were harvested at 24 h or 48 h post-transfection, the silencing efficiency was evaluated by determining the protein levels in whole-cell lysate using western blotting.

### Sample preparation for phosphoproteome

MCM2-overexpressing A549 cells, MCM2-silenced H1299 cells, and two sets of control cells (A549 and H1299 cells) were lysed using lysis buffer [12 mM sodium deoxycholate (SDC, Sigma-Aldrich) and 12 mM N-lauroylsarcosine sodium salt (SLS, MP Biomedicals) in 50 mM triethylammonium bicarbonate (TEABC, Sigma-Aldrich)] containing protease (Sigma-Aldrich) and phosphatase inhibitor cocktail (Tyrosine and Serine/Threonine phosphatase inhibitors cocktail; Bionovas). The cells were homogenized on ice using an ultrasonic homogenizer (LABSONIC M ultrasonic homogenizer; Sartorius) with 60% amplitude and 0.6 cycle duration for 1 min. Cell lysate was centrifuged at 12, 000 × g for 20 min at 4 °C. Supernatants containing the protein extract were then subjected to the protein quantification using a bicinchoninic acid Protein Assay Kit (Pierce; Thermo Fisher Scientific) according to the user manual. Protein extract was reduced with 10 mM dithiothreitol (DTT, WAKO) at room temperature for 30 min, and carbamidomethylated with 55 mM iodoacetamide (catalog no. IOD500.10; BioShop) at room temperature in the dark for 30 min. Alkylated proteins were digested with endopeptidase Lys-C (1:100 w/w) (WAKO) for 2 h followed by sequencing grade modified trypsin (1:100 w/w) (Thermo Fisher Scientific) overnight at room temperature. The trypsin reaction was inactivated by acidified the peptide solution to a pH < 3 using trifluoroacetic acid (TFA, Sigma-Aldrich). For detergent removal, the acidified peptide solution was combined with an equal volume of ethyl acetate (Sigma-Aldrich) and agitated vigorously for 1 min, followed by centrifugation at 15,700 × g for 2 min to separate the aqueous and organic phases. The sample from the aqueous phase was dried using a centrifugal evaporator and then subjected to desalting using Styrenedivinylbenzene Empore disk membranes (SDB-XC) StageTips (catalog no. 2340; 3 M) and eluted in a buffer containing 0.1% (v/v) TFA and 80% (v/v) acetonitrile (ACN)^42^.

### Dimethyl labeling of peptides for phosphoproteome

The stable isotope dimethyl labeling involves the formation of a Schiff base via the reaction of formaldehyde with the primary amines, which are then reduced by cyanoborohydride^67^. The digested peptides of two controls, MCM2-overexpressed and MCM2-silenced samples were dried using a centrifugal evaporator and reconstituted separately with 100 mM TEABC. Each control sample was labeled with 4% formaldehyde-*H*_2_ (37% Formaldehyde solution; Sigma-Aldrich). MCM2-overexpressed and MCM2-silenced samples were labeled with 4% formaldehyde-*D*_2_ (20% Formaldehyde-^13^C, d_2_ solution; Sigma-Aldrich) separately. 4 μL of 600 mM sodium cyanoborohydride (NaBH_3_CN, Sigma-Aldrich) were then added to each sample solution and incubated for 1 h at room temperature. The reaction was inactivated by adding 16 μl of 1% (v/v) ammonia solution (WAKO) and acidified the peptides using 10% (v/v) formic acid (WAKO) to a pH < 3. The H_2_-labeled A549 control and H1299 control were combined with the D_2_ labeled MCM2-overexpressed and MCM2-silenced samples at 1:1 ratio, respectively. These two combined mixtures were subjected to desalting as described previously.

### Phosphopeptide enrichment

The phosphopeptides were enriched by using hydroxy acid-modified metal oxide chromatography (HAMMOC), where home-made lactic acid-modified titania MOC tips were prepared by packing 0.5 mg titansphere beads (Titansphere TiO 10 μm; GL Sciences) into 10 μL C8 StageTips and equilibrated with solution A containing 0.1% TFA, 80% ACN, and 300 mg/mL of lactic acid (WAKO) prior to the sample loading^23,42^. Each 200 μg of the desalted peptide mixture (formaldehyde-*H*_2_ and formaldehyde-*D*_2_ labeled) was mixed with an equal volume of solution A and loaded onto the lactic acid-modified titania MOC tips (100 μg peptides/tip). After successive washing with solution A and solution B (0.1% TFA and 80% ACN), the phosphopeptides were eluted by 0.5% and 5% (v/v) piperidine (WAKO). The eluate was acidified with 10% (v/v) TFA, desalted with SDB-XC StageTip^41^ and vacuum dried as described previously. For each profile, two independent batches of biological samples were prepared. The phosphopeptides were resuspended in 0.5% TFA and subjected to nanoliquid chromatography (nanoLC)−MS/MS analysis.

### NanoLC−MS/MS analysis for MCM2 phosphoproteome

NanoLC-MS/MS was performed on a LTQ-Orbitrap XL (Thermo Elrctron), equippeded with a nanoACQUITY UPLC system (Waters). Peptide mixtures were loaded onto a 2 cm × 180 μm capillary trap column and then separated in a 75 μm ID, 25 cm length C18 column BEH nanoACQUITY at a flow rate of 300 nL/min, where mobile phases was A [0.1% formic acid (FA)] and B (0.1% FA/80% ACN). A linear gradient of 10–40% B in 90 min and 40–85% B in 10 min was employed throughout this study. Mass spectra from survey full scans were acquired on the Orbitrap (*m/z* 300–1500). The resolution of the instrument was set to 60000 at *m*/*z* 400 with an automated gain control (AGC) value of 10^6^. The top ten most-intense precursor ions were selected from the MS scan for subsequent collision-induced dissociation MS/MS scan by ion trap (AGC target at 7000). For each biological sample, duplicate nanoLC-MS/MS analyses were performed. Two biological replicates of overexpressed-MCM2 and four biological replicates of silenced-MCM2 (including two biological replicates of siMCM2–2 and two biological replicates of siMCM2-3) were performed (Supplementary Table S11). Two technical replicates were performed for each biological replicate.

### Sample preparation for proteome

MCM2-silenced H1299 cells and siRNA H1299 cells were lysed by lysis buffer [1% (v/v) sodium dodecyl sulfate (SDS, Bioman), 50 mM Tris-HCl (Bioman), 10% (v/v) glycerol (Sigma-Aldrich)] containing protease inhibitor cocktail (Bioman) using homogenizer as previously described. 1 M TEABC was added to the protein samples to make a final concentration of 50 mM and adjust the pH to about 8.5. The protein samples were reduced using 5 mM tris(2-carboxyethyl)phosphine hydrochloride (Sigma-Aldrich) at 37 °C for 30 min and alkylated using 5 mM iodoacetamide at room temperature in the dark for 30 min. Gel-assisted protein digestion was applied to obtain peptides. Acrylamide/bis (acrylamide) (40%, v/v, 37.5:1), 10% (w/w) ammonium persulfate (APS), and tetramethylethylenediamine (TEMED) were mixed with the protein solution until the solution polymerized into a gel (Protein: Acrylamide: APS: TEMED = 14:5:0.3:0.3, (v/v)). The gel was cut into small pieces and washed continuously with 25 mM TEABC and 25 mM TEABC/50% (v/v) acetonitrile (ACN, Thermo Fisher Scientific) until no bubbles were visible. The gel pieces were further dehydrated with 100% ACN and dried completely using a centrifugal evaporator (CVE-2000; Eyela). 25 mM TEABC was added to rehydrate the gel and trypsin (protein: trypsin = 10:1, w/w) for digestion. An additional volume of 25 mM TEABC was added to ensure the gels were completely covered by the solution. The samples were incubated in a water bath at 37 °C for 16 h. Peptides were extracted from the gel with 0.1% (v/v) TFA, 50% ACN/0.1% (v/v) TFA, and 100% ANC sequentially by vigorous vortexing. The extracted peptide solution was dried using a centrifugal evaporator.

### Isobaric tags for relative and absolute quantitation (iTRAQ) labeling

The peptides were resuspended in dissolution buffer to reach a final concentration of 1–1.5 μg/μL. Each vial of iTRAQ reagent was dissolved in 70 μL of absolute ethanol. 100 μg of peptide from different sample was used for each iTRAQ labeling. Peptides from the two biological replicates of siRNA control H1299 cells were labeled with iTRAQ reagent 114 and iTRAQ reagent 115; peptides from the two biological replicates of MCM2-silenced H1299 cells were labeled with iTRAQ reagent 116 and iTRAQ reagent 117. The labeling process was performed at room temperature using a continuous gentle vortex for 1 h. All labeled peptides were combined into 1:1:1:1 ratio and dried using a centrifugal evaporator.

### Strong cation exchange (SCX) chromatography

The labeled peptides were dissolved in 2 mL of buffer A [5 mM KH2PO4 and 25% (v/v) ACN, pH 3] and fractionated by SCX chromatography using a 2.1 × 200 mm PolySULFOETHYL A column containing 5 μm particles with 300 Å pore size (Poly LC). The peptides were fractionated using a flow rate of 200 μL/min and a sequential gradient of 0–25% buffer B [5 mM KH2PO4, 350 mM KCl and 25% (v/v) ACN, pH3] for 30 min, 25–100% buffer B for 20 min, 100% buffer B for 10 min, 100–0% buffer B for 5 min, and 100% buffer A for 10 min. The eluate was monitored by measuring the absorbance of the peptide bond at 214 nm. Eluates were collected every minute and dried using a centrifugal evaporator.

### Peptide desalting by ZipTip pipet tips

ZipTip pipet tips (Millipore, Bedford) were used for desalting the 20 fractionated iTRAQ labeled samples individually. The peptides were resuspended in 20 μL of 0.1% (v/v) TFA, and the pH value was adjusted to a pH < 3 by 10% (v/v) TFA. The tips were wetted using 100% ACN and 50% (v/v) ACN/0.1% (v/v) TFA and were equilibrated using 0.1% (v/v) TFA. The peptide solution was aspirated and dispensed 20 times to bind the peptides to the ZipTip pipet tips. Bound peptides were then washed using 0.1% (v/v) TFA. The peptides were eluted using 50% (v/v) ACN/0.1% (v/v) TFA and were dried using a centrifugal evaporator.

### NanoLC−MS/MS analysis for siMCM2 proteome

The MS analyses were conducted by Academia Sinica Common Mass Spectrometry Facilities. NanoLC-MS/MS analysis was performed on a nanoAcquity system (Waters) connected to an LTQ-Orbitrap XL hybrid mass spectrometer (Thermo Electron) equipped with a nanospray interface (Proxeon). Peptide mixtures were loaded onto a 75 μm ID, 25 cm length C18 BEH column (Waters) packed with 1.7 μm particles with a pore with of 130 Å and were separated using a segmented gradient in 90 min from 5% to 40% solvent B (ACN with 0.1% FA) at a flow rate of 300 nl/min and a column temperature of 35 °C. Solvent A was 0.1% FA in water. The LTQ-Orbitrap XL hybrid mass spectrometer was operated in positive ionization mode. The MS survey scan for all experiments was performed in the FT cell recording a window between 350 and 1600 *m/z*. The resolution was set to 60000 at *m/z* 400 and the automatic gain control (AGC) was set to 1000000 ions. The *m/z* values triggering MS/MS were put on an exclusion list for 90 s. In all cases, one microscan was recorded. For HCD, the applied acquisition method consisted of a survey scan to detect the peptide ions followed by a maximum of three MS/MS experiments of the three most intense signals exceeding a minimum signal of 5000 in survey scans. For MS/MS, we used a resolution of 7500, an isolation window of 3 *m/z* and a target value of 100000 ions, with maximum accumulation times of 500 ms. Fragmentation was performed with normalized collision energy of 45% and an activation time of 30 ms.

### Phosphopeptide identification and phosphosite quantification

Raw LC-MS/MS spectral information was submitted to MaxQuant software version 1.5.0.30 (http://maxquant.org). Peptide identification was performed using Andromeda Search engine against Swiss-Prot human database (September 2014, reviewed) allowing a maximum of two missed cleavage sites^43,68,69^. Search criteria were trypsin specificity, fixed modification as carbamidomethylation, and variable modifications as oxidation and phosphorylation. Precursor mass tolerance was set at 10 ppm, and fragment ion tolerance at 20 ppm. A target-decoy search strategy was used in this study. Only peptide satisfying all the following criteria were considered as qualified peptides and subjected to further analyses: (i) the peptide is considered as confidently identified (FDR < 0.01); (ii). phosphorylation sites are considered localized at a site localization probability >0.75; (iii) the peptide is unique for protein identification. All the spectra and the related information were submitted to ProteomeXChange (http://www.proteomexchange.org/, Project accession PXD002736) and can be inspected by PRIDE Inspector.

### Protein identification and quantification

The MS/MS spectral information was submitted to Proteome Discoverer version 1.4.1.14, Thermo Fisher Scientific. The data files were combined and searched against the Swiss-Prot human database allowing a maximum of two missed cleavage sites. Search criteria were trypsin specificity, variable modification as carbamidomethyl (C), oxidation (M), iTRAQ4plex (K), and iTRAQ4plex (N-term). Precursor mass tolerance was set to 10 ppm, and the fragment mass tolerance was set to 50 mmu to prevent precursor interference. The strict target false discovery rate (FDR) of the decoy database search was set at 0.01, and the relaxed target FDR was set at 0.05. The intensity of peptide assigned by corresponding iTRAQ reporter ions (*m/z* = 114, 115, 116, and 117) was extracted using Proteome Discoverer. Only peptides satisfying all the following criteria were considered as qualified peptides and subjected to further analyses: (i) the peptide is labeled with iTRAQ tags; (ii) the peptide is considered as confidently identified (FDR < 0.01); and (iii) the peptide is unique for protein identification. The normalization was performed according to the assumption that the abundances of peptides labeled with different iTRAQ were equal and all log ratios of the peptides between the siMCM2 treatment and control were normally distributed^69^. For peptides labeled with iTRAQ tag X, the peptide abundances were multiplied by normalization factor N_X_, which is expressed as

$${{\rm{N}}}_{{\rm{X}}}={2}^{-{\rm{MX}}}$$

where MX denotes the median of all log_2_ transformed iTRAQ ratios, which represents the relative peak intensity of the iTRAQ tag X signature ion *m/z* 114, 115, 116, or 117 to the iTRAQ signature ion *m/z* 114. Normalized peptide iTRAQ signals were used for calculating protein abundance.

All the spectra and the related information were submitted to ProteomeXChange (http://www.proteomexchange.org/, Project accession PXD003743) and can be inspected by PRIDE Inspector.

### Functional annotation

The protein-GO term association was obtained from Uniprot and the protein complex data were downloaded from CORUM^70^. The Fisher’s exact test with the Benjamini-Hochberg multiple testing correction was used to identify over-represented GO terms and protein complexes. The all human proteins annotated by GO and curated by CORUM were used as the background set for GO and protein complex enrichment analyses, respectively. The over-represented GO terms were visualized as a network using the EnrichmentMap Cytoscape App^71^ and enhanceGraphics^72^.

### Cell proliferation assays

Cell viability was performed by MTT and MTS. Cells were seeded at 4000 cells per 96-well plate after 24-h transfection. 3-(4,5-Dimethylthiazol-2-yl)-2,5-diphenyltetrazolium bromide (MTT, Sigma) or 3-(4,5-dimethylthiazol-2-yl)-5-(3-carboxymethoxyphenyl)-2-(4-sulfophenyl)-2H-tetrazolium, inner salt (MTS, Promega) was dissolved in PBS (5 mg/mL or 20 mg/mL). The MTT or MTS solution was added at 24 h and 48 h post-transfection and incubated for either 1 or 3 h at 37 °C with 5% CO_2_. The MTT and MTS assays were measured at 570 nm or 490 nm using an ELISA reader (BioRad). Cells were collected 24 h after transfection and 500 cells were subsequently seeded into a six-well plate for colony formation. Colonies were allowed to form for 8 d, after which they were fixed with 100% methanol (Sigma) and stained with crystal violet (0.2 mg/mL) for 15 min.

### Cell cycle analysis

Cells were collected, fixed in 70% ethanol, and stored at −20 °C overnight. The cells were washed with PBS and resuspended in PBS containing 100 μg/mL RNase A and 0.1% Triton X-100, and incubated at 37 °C for 1 h. Cells were stained with 5 μg/mL propidium iodide (Santa Cruz) for 15 min in the dark. The DNA content of the cells was analyzed using a FACSCanto instrument (BD Biosciences Immunocytometry Systems). Ten thousand cells were collected for each measurement in a triplicate experiment. The percentage of cells in different phases of the cell cycle was analyzed using ModFit LT (Verity Software House).

### Cell migration assays

At 48 h post-transfection, 3 × 10^4^ cells with serum-free medium were loaded into the inserts, and medium containing 10% FBS was loaded into the lower compartments of an 8-μM pore size Transwell plate (Corning). The cells were incubated at 37 °C with 5% CO_2_ for 6 h. The cells were fixed for 30 min with 100% methanol and stained with 0.1% crystal violet for 30 min. Cotton swabs were used to remove cells from the upper side of the inserts. Images of five different microscope fields of each insert were captured and the cells were counted.

### Site-directed mutagenesis

PCR was performed to generate pCMV-HA plasmids (Invitrogen) with a full-length HMGA1 sequence by using the primer pair as follows: HMGA1-Forward, 5′-AAAGAATTCGCCACCATGAGTGAGTCGAGCTCG-3′; HMGA1-Reverse, 5′-AAATTGCGGCCGCTCACTGCTCCTCCTCCGAGGACT-3′. The amplified DNA fragment was digested with restriction enzymes *Eco*RI and *Not*I (Thermo Fisher Scientific). Synthesis of the mutant strand was performed by PCR using the plasmid pCMV-HA-HMGA1 as template in the presence of Phusion high-fidelity DNA polymerase (Finnzymes). Primers containing the desired mutation, as follows: *HMGA1*-S99A-Forward, 5′-GGAGGGCATCGCGCAGGAGTC-3′; *HMGA1*-S99A-Reverse, 5′-GACTCCTGCGCGATGCCCTCC-3′; *HMGA1*-S99E-Forward, 5′-GAGGAGGGCATCGAGCAGGAGTCCTCG-3′; *HMGA1*-S99E-Reverse, 5′-CGAGGACTCCTGCTCGATGCCCTCCTC-3′. Parameters were chosen according to the QuikChange site-directed mutagenesis kit (Stratagene). 1 μL of the restriction enzyme *Dpn*I (Merck) was added and incubated at 37 °C for 1 h, after which 10 μL of the *Dpn*I-treated DNA from each amplification reaction was transformed into the *E. coli* strain DH5α. The plasmid DNA was sequenced at the DNA Sequencing Facility (Genomics BioSci & Tech.).

### Immunoblot analysis

Protein extracts were separated by SDS-PAGE and transferred onto a PVDF membrane (Millipore) and immunoblotted with antibodies. The membrane was blocked in 5% non-fat milk/TBST and incubated overnight with primary antibody diluted in blocking buffer at 4 °C: rabbit anti-MCM2 (GeneTex; 1:1000), rabbit anti-HMGA1 (abcam; 1:1000) and mouse anti-Actin (Millipore; 1:5000). The membrane was then treated with secondary HRP-conjugated antibody anti-rabbit or anti-mouse IgG (Sigma-Aldrich; 1:100000) for 2 h at room temperature. Images were acquired using ECL substrate (BioRad) and FluorChem M (ProteinSimple).

### Statistical analysis

Results are expressed as the mean ± standard deviation. The Student’s *t* test was used to analyze the cell viability assay, colony formation assay, migration assay and flow cytometry. The differences between groups were considered to be statistically significant when *P* < 0.05.

### Data availability

All the spectra and the related information were submitted to ProteomeXChange (http://www.proteomexchange.org/, Project accession PXD002736 and PXD003743) and can be inspected by PRIDE Inspector.

## Electronic supplementary material

Supplementary Figures

Supplementary Table S1

Supplementary Table S2

Supplementary Table S3

Supplementary Table S4

Supplementary Table S5

Supplementary Table S6

Supplementary Table S7

Supplementary Table S8

Supplementary Table S9

Supplementary Table S10

Supplementary Table S11

## References

[1] Siegel RL, Miller KD, Jemal A (2016). Cancer statistics, 2016. CA Cancer J Clin.

[2] Crino L, Weder W, van Meerbeeck J, Felip E, Group EGW (2010). Early stage and locally advanced (non-metastatic) non-small-cell lung cancer: ESMO Clinical Practice Guidelines for diagnosis, treatment and follow-up. Ann Oncol.

[3] Cheung CHY, Juan HF (2017). Quantitative proteomics in lung cancer. J Biomed Sci.

[4] Croce CM (2008). Oncogenes and cancer. N Engl J Med.

[5] Collins K, Jacks T, Pavletich NP (1997). The cell cycle and cancer. Proc Natl Acad Sci USA.

[6] Macheret M, Halazonetis TD (2015). DNA replication stress as a hallmark of cancer. Annu Rev Pathol.

[7] Pogorelcnik B, Perdih A, Solmajer T (2013). Recent developments of DNA poisons–human DNA topoisomerase IIalpha inhibitors–as anticancer agents. Curr Pharm Des.

[8] Singh DK (2014). Human DNA ligases: a comprehensive new look for cancer therapy. Med Res Rev.

[9] Aye Y, Li M, Long MJ, Weiss RS (2015). Ribonucleotide reductase and cancer: biological mechanisms and targeted therapies. Oncogene.

[10] Simon NE, Schwacha A (2014). The Mcm2-7 replicative helicase: a promising chemotherapeutic target. Biomed Res Int.

[11] Blow JJ, Gillespie PJ (2008). Replication licensing and cancer–a fatal entanglement?. Nat Rev Cancer.

[12] Diffley JF (2004). Regulation of early events in chromosome replication. Curr Biol.

[13] Bell SP, Dutta A (2002). DNA replication in eukaryotic cells. Annu Rev Biochem.

[14] Evrin C (2009). A double-hexameric MCM2-7 complex is loaded onto origin DNA during licensing of eukaryotic DNA replication. Proc Natl Acad Sci USA.

[15] Lei M, Tye BK (2001). Initiating DNA synthesis: from recruiting to activating the MCM complex. J Cell Sci.

[16] Remus D (2009). Concerted loading of Mcm2-7 double hexamers around DNA during DNA replication origin licensing. Cell.

[17] Hubbard MJ, Cohen P (1993). On target with a new mechanism for the regulation of protein phosphorylation. Trends Biochem Sci.

[18] Humphrey SJ, James DE, Mann M (2015). Protein Phosphorylation: A Major Switch Mechanism for Metabolic Regulation. Trends Endocrinol Metab.

[19] Hornbeck PV (2015). PhosphoSitePlus, 2014: mutations, PTMs and recalibrations. Nucleic Acids Res.

[20] Hu CW (2015). Temporal Phosphoproteome Dynamics Induced by an ATP Synthase Inhibitor Citreoviridin. Mol Cell Proteomics.

[21] Lundby A (2012). Quantitative maps of protein phosphorylation sites across 14 different rat organs and tissues. Nat Commun.

[22] Wolschin F, Wienkoop S, Weckwerth W (2005). Enrichment of phosphorylated proteins and peptides from complex mixtures using metal oxide/hydroxide affinity chromatography (MOAC). Proteomics.

[23] Sugiyama N (2007). Phosphopeptide enrichment by aliphatic hydroxy acid-modified metal oxide chromatography for nano-LC-MS/MS in proteomics applications. Mol Cell Proteomics.

[24] Larsen MR, Thingholm TE, Jensen ON, Roepstorff P, Jorgensen TJ (2005). Highly selective enrichment of phosphorylated peptides from peptide mixtures using titanium dioxide microcolumns. Mol Cell Proteomics.

[25] Maine GT, Sinha P, Tye BK (1984). Mutants of S. cerevisiae defective in the maintenance of minichromosomes. Genetics.

[26] Bochman ML, Schwacha A (2007). Differences in the single-stranded DNA binding activities of MCM2-7 and MCM467: MCM2 and MCM5 define a slow ATP-dependent step. J Biol Chem.

[27] Yang J (2006). Prognostic significance of MCM2, Ki-67 and gelsolin in non-small cell lung cancer. BMC Cancer.

[28] Ha SA (2004). Cancer-associated expression of minichromosome maintenance 3 gene in several human cancers and its involvement in tumorigenesis. Clin Cancer Res.

[29] Kikuchi J (2011). Minichromosome maintenance (MCM) protein 4 as a marker for proliferation and its clinical and clinicopathological significance in non-small cell lung cancer. Lung Cancer.

[30] Fujioka S (2009). Expression of minichromosome maintenance 7 (MCM7) in small lung adenocarcinomas (pT1): Prognostic implication. Lung Cancer.

[31] Vigouroux C (2015). Methyl(R217)HuR and MCM6 are inversely correlated and are prognostic markers in non small cell lung carcinoma. Lung Cancer.

[32] Wu X, Ruan L, Yang Y, Mei Q (2016). Identification of crucial regulatory relationships between long non-coding RNAs and protein-coding genes in lung squamous cell carcinoma. Mol Cell Probes.

[33] Pape T (2003). Hexameric ring structure of the full-length archaeal MCM protein complex. EMBO Rep.

[34] Samel SA (2014). A unique DNA entry gate serves for regulated loading of the eukaryotic replicative helicase MCM2-7 onto DNA. Genes Dev.

[35] Bochman ML, Schwacha A (2008). The Mcm2-7 complex has *in vitro* helicase activity. Mol Cell.

[36] Feng J (2015). PTEN Controls the DNA Replication Process through MCM2 in Response to Replicative Stress. Cell Rep.

[37] Montagnoli A (2006). Identification of Mcm2 phosphorylation sites by S-phase-regulating kinases. J Biol Chem.

[38] Liu M (2013). MCM2 expression levels predict diagnosis and prognosis in gastric cardiac cancer. Histol Histopathol.

[39] Meng MV (2001). Minichromosome maintenance protein 2 expression in prostate: characterization and association with outcome after therapy for cancer. Clin Cancer Res.

[40] Ramnath N (2001). MCM2 is an independent predictor of survival in patients with non-small-cell lung cancer. J Clin Oncol.

[41] Kyono Y, Sugiyama N, Imami K, Tomita M, Ishihama Y (2008). Successive and selective release of phosphorylated peptides captured by hydroxy acid-modified metal oxide chromatography. J Proteome Res.

[42] Rappsilber J, Mann M, Ishihama Y (2007). Protocol for micro-purification, enrichment, pre-fractionation and storage of peptides for proteomics using StageTips. Nat Protoc.

[43] Cox J, Mann M (2008). MaxQuant enables high peptide identification rates, individualized p.p.b.-range mass accuracies and proteome-wide protein quantification. Nat Biotechnol.

[44] Cooper TA, Wan L, Dreyfuss G (2009). RNA and disease. Cell.

[45] Sveen A, Kilpinen S, Ruusulehto A, Lothe RA, Skotheim RI (2016). Aberrant RNA splicing in cancer; expression changes and driver mutations of splicing factor genes. Oncogene.

[46] Venables JP (2009). Cancer-associated regulation of alternative splicing. Nat Struct Mol Biol.

[47] Ghigna C, Valacca C, Biamonti G (2008). Alternative splicing and tumor progression. Curr Genomics.

[48] Bertoli C, Skotheim JM, de Bruin RA (2013). Control of cell cycle transcription during G1 and S phases. Nat Rev Mol Cell Biol.

[49] Szklarczyk D (2015). STRINGv10: protein-protein interaction networks, integrated over the tree of life. Nucleic Acids Res.

[50] Thomae AW (2008). Interaction between HMGA1a and the origin recognition complex creates site-specific replication origins. Proc Natl Acad Sci USA.

[51] Norseen J (2008). RNA-dependent recruitment of the origin recognition complex. EMBO J.

[52] Thomae AW (2011). Different roles of the human Orc6 protein in the replication initiation process. Cell Mol Life Sci.

[53] Wang DZ, Ray P, Boothby M (1995). Interleukin 4-inducible phosphorylation of HMG-I(Y) is inhibited by rapamycin. J Biol Chem.

[54] Sgarra R (2004). Nuclear phosphoproteins HMGA and their relationship with chromatin structure and cancer. FEBS Lett.

[55] Zou Y, Wang Y (2005). Tandem mass spectrometry for the examination of the posttranslational modifications of high-mobility group A1proteins: symmetric and asymmetric dimethylation of Arg25 in HMGA1a protein. . Biochemistry.

[56] Zhang Q, Wang Y (2008). High mobility group proteins and their post-translational modifications. Biochim Biophys Acta.

[57] Liu Y (2013). MCM-2 is a therapeutic target of Trichostatin A in colon cancer cells. Toxicol Lett.

[58] Zhang X (2015). MCM2 is a therapeutic target of lovastatin in human non-small cell lung carcinomas. Oncol Rep.

[59] Harsha HC, Pandey A (2010). Phosphoproteomics in cancer. Mol Oncol.

[60] Brognard J, Hunter T (2011). Protein kinase signaling networks in cancer. Curr Opin Genet Dev.

[61] Vijayraghavan S, Schwacha A (2012). The eukaryotic Mcm2-7 replicative helicase. Subcell Biochem.

[62] Dang HQ, Li Z (2011). The Cdc45.Mcm2-7.GINS protein complex in trypanosomes regulates DNA replication and interacts with two Orc1-like proteins in the origin recognition complex. J Biol Chem.

[63] Stoeber K (2001). DNA replication licensing and human cell proliferation. J Cell Sci.

[64] Catez F, Hock R (2010). Binding and interplay of HMG proteins on chromatin: lessons from live cell imaging. Biochim Biophys Acta.

[65] Zhang ZH (2015). WNK1 is involved in Nogo66 inhibition of OPC differentiation. Mol Cell Neurosci.

[66] Edberg DD, Bruce JE, Siems WF, Reeves R (2004). *In vivo* posttranslational modifications of the high mobility group A1a proteins in breast cancer cells of differing metastatic potential. Biochemistry.

[67] Boersema PJ, Raijmakers R, Lemeer S, Mohammed S, Heck AJ (2009). Multiplex peptide stable isotope dimethyl labeling for quantitative proteomics. Nat Protoc.

[68] Cox J (2011). Andromeda: a peptide search engine integrated into the MaxQuant environment. J Proteome Res.

[69] Wu YH (2013). Quantitative proteomic analysis of human lung tumor xenografts treated with the ectopic ATP synthase inhibitor citreoviridin. PLoS One.

[70] Ruepp A (2010). CORUM: the comprehensive resource of mammalian protein complexes–2009. Nucleic Acids Res.

[71] Merico D, Isserlin R, Stueker O, Emili A, Bader GD (2010). Enrichment map: a network-based method for gene-set enrichment visualization and interpretation. PLoS One.

[72] Morris JH, Kuchinsky A, Ferrin TE, Pico AR (2014). enhancedGraphics: a Cytoscape app for enhanced node graphics. F1000Res.

