# Supplementary material for: MCM2-regulated functional networks in lung cancer by multi-dimensional proteomic approach
Source: Sci Rep. 2017 Oct 16;7:13302. doi: 10.1038/s41598-017-13440-x (PMC5643318; doi:10.1038/s41598-017-13440-x)
Supplement: Supplementary file 1 — Supplementary Figures [file 41598_2017_13440_MOESM1_ESM.pdf]

Supplementary Information

MCM2-regulated functional networks in lung cancer by multi-dimensional proteomic approach

Chantal Hoi Yin Cheung[1], Chia-Lang Hsu[2], Kai-Pu Chen[3], Siao-Ting Chong[1], Chang-Hsun Wu, Hsuan-Cheng Huang[4,*], Hsueh-Fen Juan[1, 2, 3,*]

[1]Institute of Molecular and Cellular Biology, National Taiwan University, Taipei 10617, Taiwan.

[2]Department of Life Science, National Taiwan University, Taipei 10617, Taiwan.

[3]Graduate Institute of Biomedical Electronics and Bioinformatics, National Taiwan University, Taipei 10617, Taiwan.

[4]Institute of Biomedical Informatics, Center for Systems and Synthetic Biology, National Yang-Ming University, Taipei 11221, Taiwan.

The supplementary information contains the following files:

I. Supplementary Figures (a single PDF file)

- Supplementary Figure S1. Clinical significance of MCM subunit expression in lung adenocarcinoma.
- Supplementary Figure S2. MCM2 expression between cancerous and adjacent normal tissues across 14 cancer types.
- Supplementary Figure S3. Endogenous expression of MCM2 in lung cancer cells.
- Supplementary Figure S4. MCM2 protein expression upon MCM2 overexpression in A549 cells and silencing MCM2 in H1299 cells at 12 h, 24 h and 48 h time points.
- Supplementary Figure S5. Phosphoproteomic profile of pMCM2 in A549 cells.
- Supplementary Figure S6. Phosphoproteomic profile of siMCM2 in H1299 cells.
- Supplementary Figure S7. Global proteomic profile of siMCM2 in H1299 cells by iTRAQ showed high reproducibility and accuracy.
- Supplementary Figure S8. Global proteomic profile of siMCM2 in H1299 cells.
- Supplementary Figure S9. Full length western blots of HMGA1 transfected A549 and H1299 cells.

II. Supplementary Tables (separate Microsoft Excel files)

- Supplementary Table S1. All identified phosphopeptides of pMCM2 and siMCM2 phosphoproteome in NSCLC.
- Supplementary Table S2. All identified phosphosites of pMCM2 and siMCM2 phosphoproteome in NSCLC.
- Supplementary Table S3. Significantly regulated phosphosites of pMCM2 phosphoproteome in A549 cells.
- Supplementary Table S4. Significantly regulated phosphosites of siMCM2 phosphoproteome in H1299 cells.
- Supplementary Table S5. Global proteome profile of siMCM2 in H1299 cells by iTRAQ.
- Supplementary Table S6. Significantly regulated proteins of siMCM2 global proteome in H1299 cells by iTRAQ.
- Supplementary Table S7. Table for gene ontology analysis.
- Supplementary Table S8. Complex identified from functional annotation of MCM2-regulated phosphoproteome and proteome.
- Supplementary Table S9. All identified phosphosites of pMCM2 phosphoproteome in A549 cells.
- Supplementary Table S10. All identified phosphosites of siMCM2 phosphoproteome in H1299 cells.
- Supplementary Table S11. All biological replicates of identified phosphosites of siMCM2 and pMCM2 phosphoproteome in NSCLC.

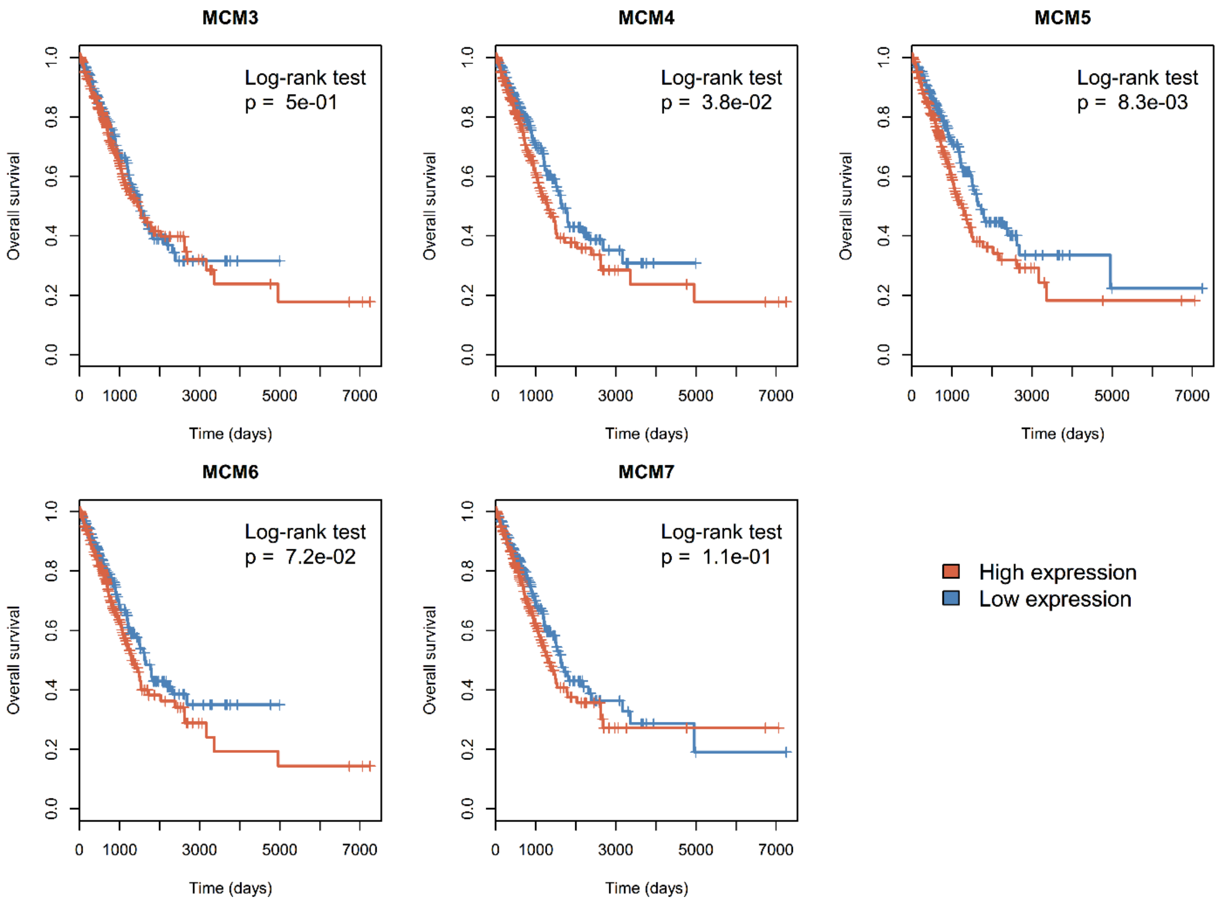

Supplementary Figure S1. Clinical significance of MCM subunit expression in lung adenocarcinoma.

Patients were stratified according to the median of the marked gene expression.

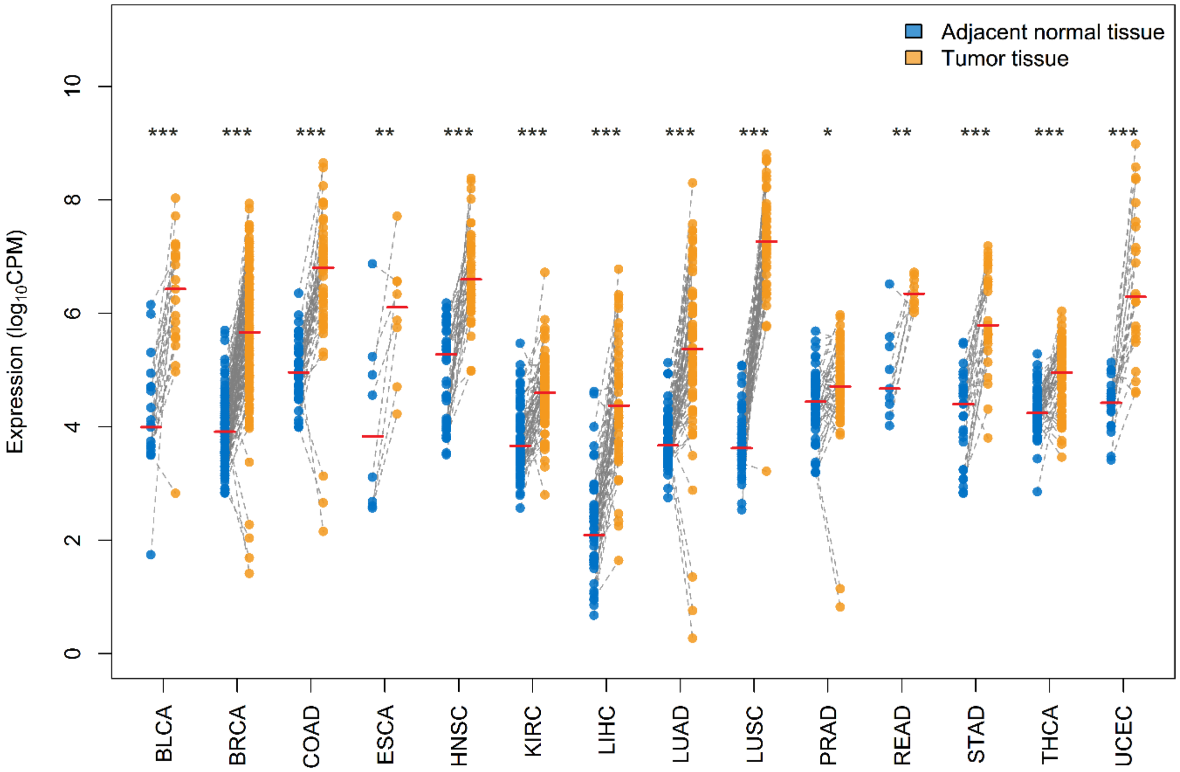

Supplementary Figure S2. MCM2 expression between cancerous and adjacent normal tissues across 14 cancer types.

The MCM2 expression in the matched tumor and adjacent normal tissues were shown. All samples were collected from TCGA. The significances were assessed by the paired *t*-test (* < 0.05, ** < 0.01, and *** < 0.001). BLCA: Bladder urothelial carcinoma; BRCA: Breast invasive carcinoma; COAD: Colon adenocarcinoma; ESCA: Esophageal carcinoma; HNSC: Head and Neck squamous cell carcinoma; KIRC: Kidney renal clear cell carcinoma; LIHC: Liver hepatocellular carcinoma; LUAD: Lung adenocarcinoma; LUSC: Lung squamous cell carcinoma; PRAD: Pancreatic adenocarcinoma; READ: Rectum adenocarcinoma; STAD: Stomach adenocarcinoma; THCA: Thyroid carcinoma; UCEC: Uterine corpus endometrial carcinoma. CPM: Counts per million.

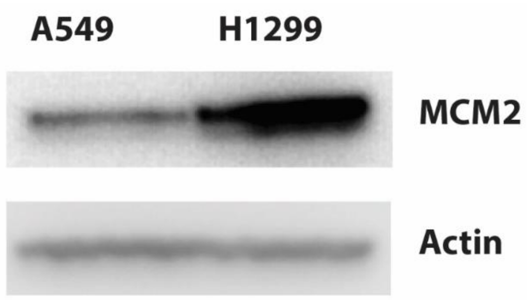

Supplementary Figure S3. Endogenous expression of MCM2 in lung cancer cells. Protein (30 μg) were separated using a 10% SDS polyacrylamide gel and transferred onto a PVDF membrane. The membrane was incubated with the following primary antibody diluted in blocking buffer at 4°C overnight: rabbit anti-MCM2 (1:1000), and mouse anti-Actin (1:5000) followed by incubation with appropriate horseradish peroxidase-labeled secondary antibody (1:100000) for 2 hours at room temperature. Signal was developed with Clarity Western ECL Substrate Kit and the images were captured using FluroChem M. The western blotting revealed a significantly higher expression of endogenous MCM2 protein in H1299 cells relative to A549 cells.

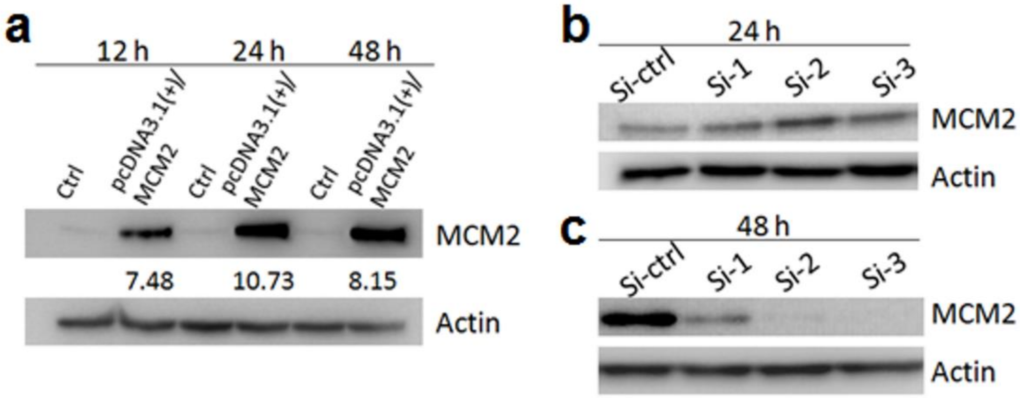

Supplementary Figure S4. MCM2 protein expression upon MCM2 overexpression in A549 cells and silencing MCM2 in H1299 cells at 12 h, 24 h and 48 h time points. (**a**) A549 cells were transfected with 1 µg pcDNA3.1(+)/MCM2 or control vector. Cells were harvested at 12 h, 24 h and 48 h post-transfection, then the protein levels were analyzed by Western blotting. Highest MCM2 protein expression level of overexpressed MCM2 was at 24 h post-transfection. (**b**) and (**c**) H1299 cells were treated with three siMCM2 (si-1, si-2 and si-3) or control siRNA at a final concentration of 10nM. Cells were harvested at 24 h and 48 h, and the protein levels were analyzed by Western blotting. siMCM2 (si-2 and si-3) showed a higher knockdown efficiency at 48 h.

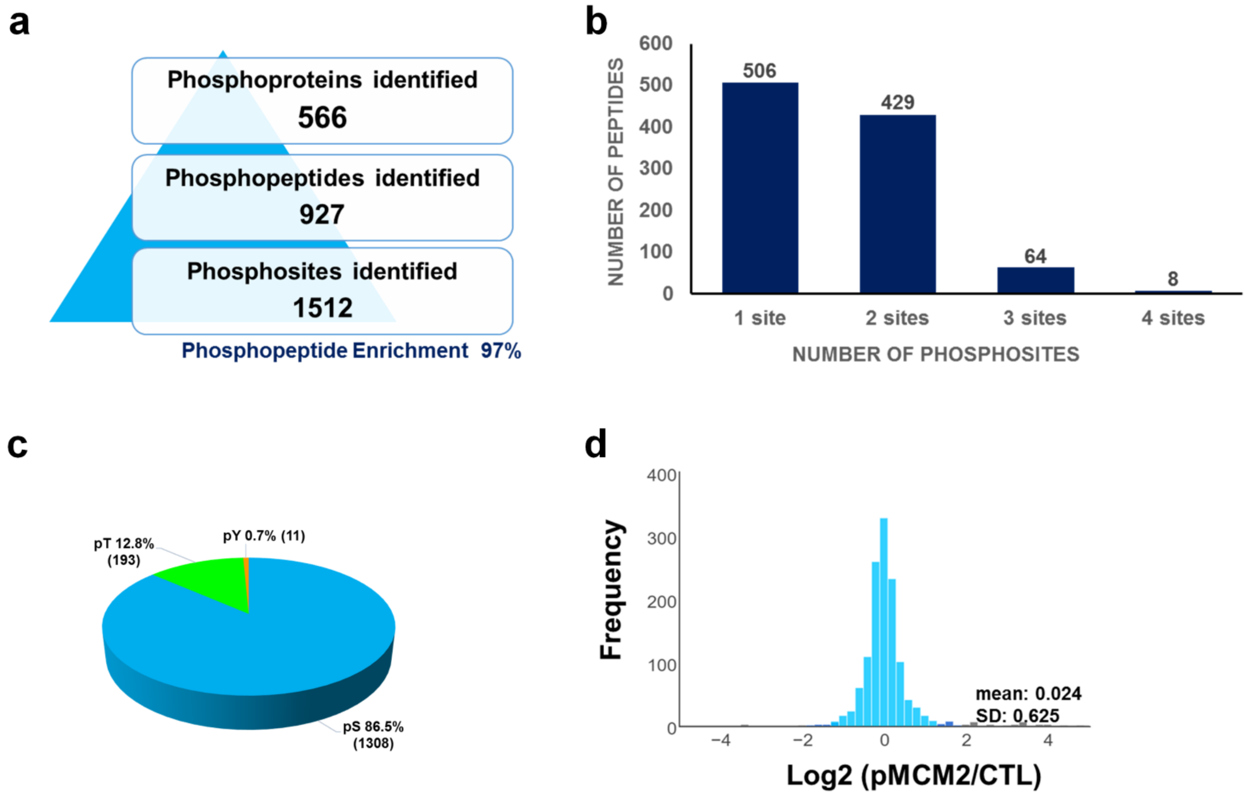

Supplementary Figure S5. Phosphoproteomic profile of pMCM2 in A549 cells. (**a**) Number of identified phosphoproteins, phosphopeptides, and phosphosites in pMCM2 A549 cells phopshoproteomic profile. (**b**) Number of singly, doubly, triply and quadruply phosphorylated peptides. (**c**) Distribution of phosphorylated serine, threonine, and tyrosine sites. Pie chart showed the distribution of phosphorylation sites on Ser (86.5%) Thr (12.8%) and Tyr (0.7%) residues. (**d**) Normal distribution of protein abundance (Log_2 ratio of pMCM2 to control) in A549 cells phosphoproteome.

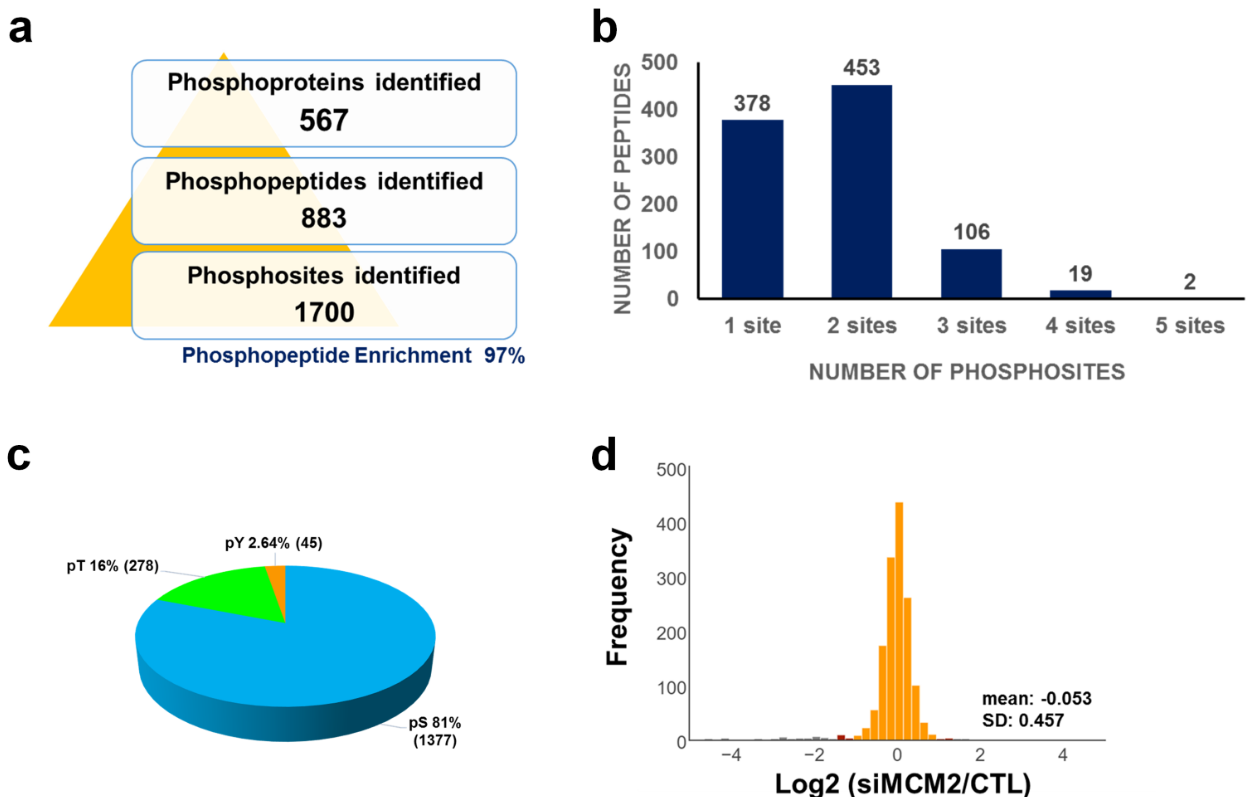

Supplementary Figure S6. Phosphoproteomic profile of siMCM2 in H1299 cells. (**a**) Number of identified phosphoproteins, phosphopeptides, and phosphosites in siMCM2 H1299 cells phopshoproteomic profile. (**b**) Number of singly, doubly, triply and quadruply phosphorylated peptides. (**c**) Distribution of phosphorylated serine, threonine, and tyrosine sites. Pie chart showed the distribution of phosphorylation sites on Ser (81%) Thr (16%) and Tyr (3%) residues. (**d**) Normal distribution of protein abundance (Log_2 ratio of siMCM2 to control) in H1299 cells phosphoproteome.

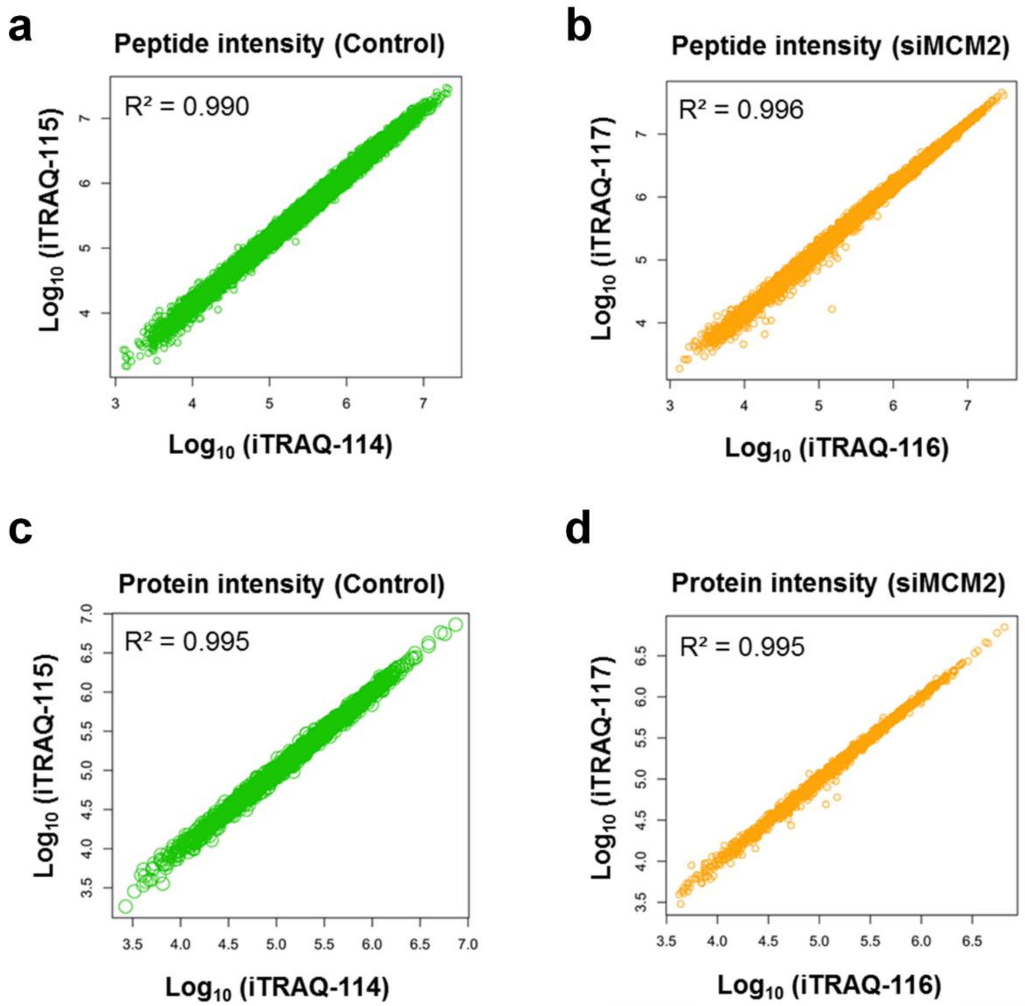

Supplementary Figure S7. Global proteomic profile of siMCM2 in H1299 cells by iTRAQ showed high reproducibility and accuracy. (**a**) Scattering plot of two peptide intensity of siRNA control replicates in H1299 cells, iTRAQ 114-labeled siRNA control-A and iTRAQ 115-labeled siRNA control-B. (**b**) Scattering plot of two peptide intensity of MCM2 silenced replicates in H1299 cells, iTRAQ 116-labeled siMCM2-A and iTRAQ 117-labeled siMCM2-B. (**c**) Scattering plot of two protein intensity of siRNA control replicates in H1299 cells, iTRAQ 114-labeled siRNA control-A and iTRAQ 115-labeled siRNA control-B. (**d**) Scattering plot of two protein intensity of MCM2 silenced replicates in H1299 cells, iTRAQ 116-labeled siMCM2-A and iTRAQ 117-labeled siMCM2-B.

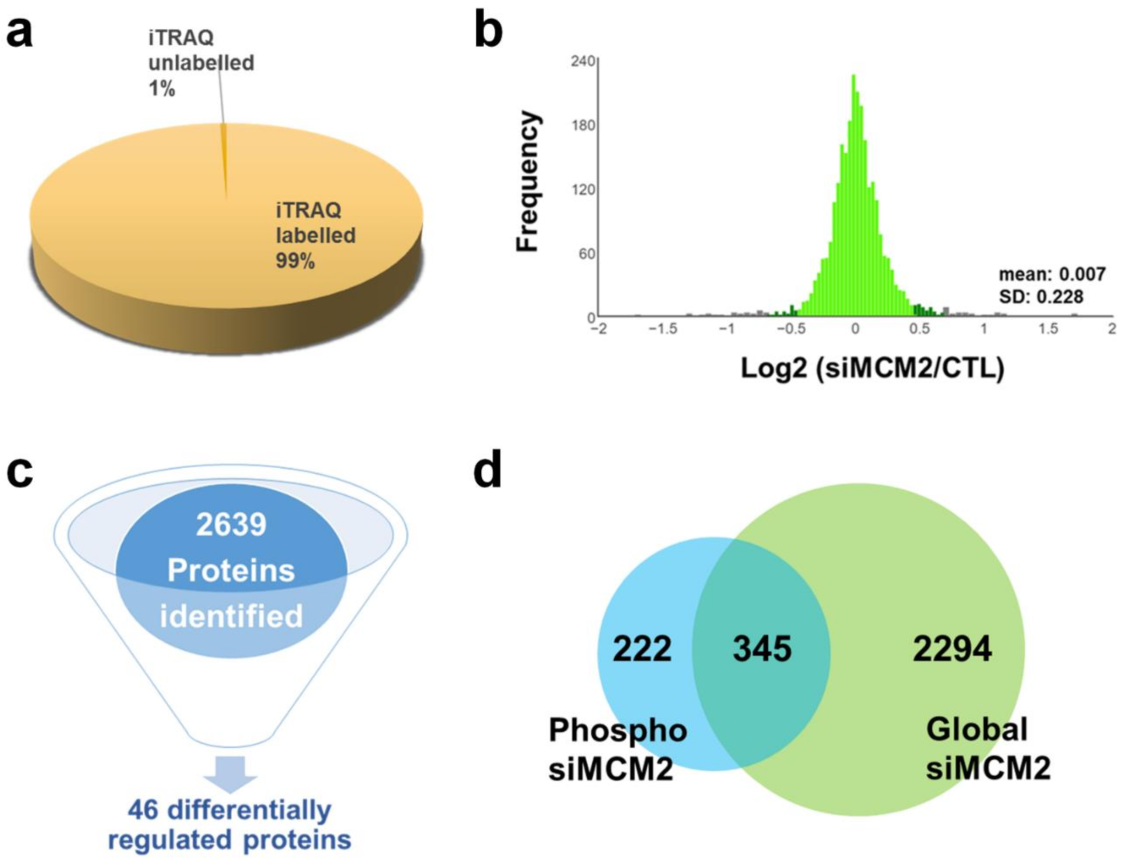

Supplementary Figure S8. Global proteomic profile of siMCM2 in H1299 cells. (**a**) iTRAQ labeling efficiency, 99.2 % of iTRAQ labeled peptides (yellow) compare to unlabeled peptides 0.8% (orange). (**b**) Normal distribution of protein abundance (Log_2 ratio of siMCM2 to control) in H1299 cells global proteomic profile. (**c**) Significantly regulated proteins in response to siMCM2 in H1299 cells, 46 differentially regulated proteins with at least 1.5 fold-changed. (**d**) 345 proteins were identified in both global proteome and phophoproteome in response to siMCM2 in H1299 cells.

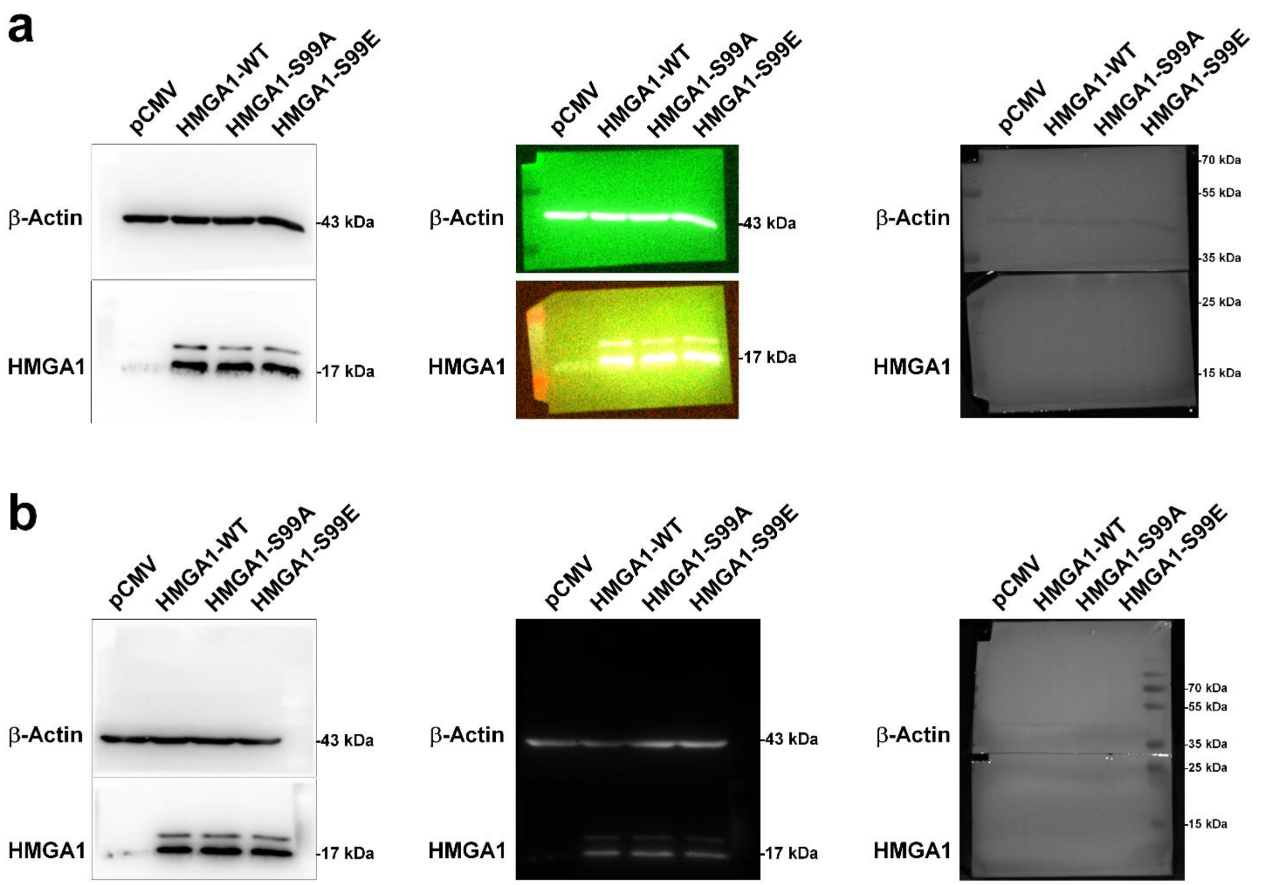

Supplementary Figure S9. Full length western blots of HMGA1 transfected A549 and H1299 cells. (**a**) Western blot showing the total HMGA1 protein expression of A549 cells 48 h after transfection of pCMV vector (pCMV), HMGA1 wild-type (HMGA1-WT), HMGA1 ^{S99A} mutants (HMGA1-S99A), and HMGA1^{S99E} mutants (HMGA1-S99E). Immunoblot analysis of HMGA1 and β-Actin protein levels (left), merged with marker (middle), bright field (right) of HMGA1 transfected A549 cells. (**b**) Western blot showing the total HMGA1 protein expression of H1299 cells 48 h after transfection of pCMV vector (pCMV), HMGA1 wild-type (HMGA1-WT), HMGA1 ^{S99A} mutants (HMGA1-S99A), and HMGA1^{S99E} mutants (HMGA1-S99E). Immunoblot analysis of HMGA1 and β-Actin protein levels (left), a single image of full length blots (middle), bright field (right) of HMGA1 transfected H1299 cells. These full length blots correspond to the

cropped images presented in Figure 7c.
